# Comparison of 6-Month Outcomes of Endovascular vs Surgical Revascularization for Patients With Critical Limb Ischemia

**DOI:** 10.1001/jamanetworkopen.2022.27746

**Published:** 2022-08-19

**Authors:** Monil Majmundar, Kunal N. Patel, Rajkumar Doshi, Mahesh Anantha-Narayanan, Ashish Kumar, Grant W. Reed, Rishi Puri, Samir R. Kapadia, Ziad A. Jaradat, Deepak L. Bhatt, Ankur Kalra

**Affiliations:** 1 Department of Cardiovascular Medicine, University of Kansas Medical Center, Kansas City; 2 Department of Internal Medicine, St Peter’s University Hospital, New Brunswick, New Jersey; 3 Department of Cardiology, St Joseph’s University Medical Center, Paterson, New Jersey; 4 Department of Cardiovascular Medicine, University of Arkansas, Little Rock; 5 Department of Internal Medicine, Cleveland Clinic Akron General, Akron, Ohio; 6 Department of Cardiovascular Medicine, Heart, Vascular, and Thoracic Institute, Cleveland Clinic, Cleveland, Ohio; 7 Division of Cardiovascular Medicine, Krannert Cardiovascular Research Center, Indiana University School of Medicine, Indianapolis; 8 Brigham and Women’s Heart and Vascular Center, Harvard Medical School, Boston, Massachusetts; 9 Cardiovascular Institute, Kalra Hospitals, New Delhi, India

## Abstract

**Importance:**

The Bypass Versus Angioplasty for Severe Ischemia of the Leg randomized controlled trial showed comparable outcomes between endovascular revascularization (ER) and surgical revascularization (SR) for patients with critical limb ischemia (CLI). However, several observational studies showed mixed results. Most of these studies were conducted before advanced endovascular technologies were available.

**Objective:**

To compare ER and SR treatment strategies for 6-month outcomes among patients with CLI.

**Design, Setting, and Participants:**

This retrospective, population-based cohort study used the Nationwide Readmissions Database to identify 66 277 patients with CLI who underwent ER or SR from January 1, 2016, to December 31, 2018. Data analyses were conducted from January 1, 2022, to February 8, 2022. A propensity score with 1:1 matching was applied. Patients with CLI who underwent ER or SR were identified, and those with missing information on the length of stay and/or younger than 18 years were excluded.

**Exposures:**

Endovascular or surgical revascularization.

**Main Outcomes and Measures:**

The primary outcome was a major amputation at 6 months. Significant secondary outcomes were in-hospital and 6-month mortality and an in-hospital safety composite of acute kidney injury, major bleeding, and vascular complication. Subgroup analysis was conducted for major amputation in high-volume centers.

**Results:**

A total of 66 277 patients were identified between 2016 and 2018 who underwent ER or SR for CLI. The Nationwide Readmissions Database does not provide racial and ethnic categories. The mean (SD) age of the cohort was 69.3 (12) years, and 62.5% of patients were male. A total of 54 546 patients (82.3%) underwent ER and 11 731 (17.7%) underwent SR. After propensity score matching, 11 106 matched pairs were found. Endovascular revascularization was associated with an 18% higher risk of major amputation compared with SR (997 of 10 090 [9.9%] vs 869 of 10 318 [8.4%]; hazard ratio, 1.18; 95% CI, 1.08-1.29; *P* = .001). However, no difference was observed in major amputation risk when both procedures were performed in high-volume centers. Endovascular revascularization and SR had similar mortality rates (517 of 11 106 [4.7%] vs 490 of 11 106 [4.4%]; hazard ratio, 1.06; 95% CI, 0.93-1.20; *P* = .39). However, the ER group had a 17% lower risk of in-hospital safety outcomes compared with the SR group (2584 of 11 106 [23.3%] vs 2979 of 11 106 [26.8%]; odds ratio, 0.83; 95% CI, 0.78-0.88; *P* < .001).

**Conclusions and Relevance:**

The results of this study suggest that ER was safer, without any difference in mortality, but ER was associated with an increased risk of major amputation compared with SR. However, the risk of major amputation was similar when both procedures were performed at high-volume centers.

## Key Points

**Question:**

What is the difference between endovascular and surgical revascularization regarding in-hospital safety, 6-month major amputation, and mortality for patients with critical limb ischemia?

**Findings:**

In this cohort study of 11 106 matched pairs, endovascular revascularization was associated with an 18% higher risk of major amputation at 6 months and a 17% lower risk of in-hospital safety outcomes compared with surgical revascularization, with no difference in mortality between the 2 groups at 6 months. The difference in major amputation outcome was not observed in high-volume centers.

**Meaning:**

Findings suggest that endovascular revascularization has a better safety profile than surgical revascularization, without any difference in mortality; hence, procedure selection should be a shared and informed decision with the patient.

## Teaser

This cohort study compares endovascular vs surgical revascularization treatment strategies regarding 6-month outcomes among propensity score–matched patients with critical limb ischemia.

## Introduction

Peripheral artery disease affects more than 230 million people worldwide, and approximately 11% of patients with the disease present with critical limb ischemia (CLI).^1,2^ Hospitalizations for CLI increased significantly from 2011 to 2017 and are associated with higher rates of all-cause readmissions (27.1% at 30 days and 56.6% at 6 months).^3,4^ Such hospitalizations cost approximately $4.2 billion annually, with an additional 30-day readmission cost of $624 million.^5^ Surgical revascularization (SR) has been the mainstay therapy for CLI for decades; however, endovascular revascularization (ER) is emerging as an effective alternative therapy for CLI management. The introduction of newer drug-eluting stents, drug-coated balloons, pedal arch reconstruction, venous arterialization, and atherectomy devices has led to a steady increase in the success rate for endovascular interventions, given its advantages of better short-term outcomes and less resource use.^3^

According to 2016 American Heart Association/American College of Cardiology guidelines, SR is preferred (Class I) for patients with a common femoral artery lesion, a long segment lesion, and lesions at multiple anatomic levels in an artery, whereas ER is considered (Class IIa) for patients with femoropopliteal disease.^6^ The recommendations are mainly based on the Bypass Versus Angioplasty for Severe Ischemia of the Leg (BASIL) trial that showed similar amputation-free survival and mortality among patients between ER and SR.^7^ However, it is not easy to draw firm conclusions from the BASIL trial because it included balloon angioplasty without drug coating in the endovascular approach. Since the trial concluded, new endovascular therapies have emerged, and there is a paucity of data comparing new ER techniques with SR. The ongoing BASIL-3 and Best Endovascular vs Best Surgical Therapy in Patients With Critical Limb Ischemia trials will provide more clarity on the choice of revascularization. With this background, we aimed to compare ER and SR treatment strategies and analyze 6-month outcomes among patients with CLI from the most recently available Nationwide Readmissions Database (NRD).

## Methods

### Data Source

For this cohort study, we obtained our study population from the NRD between January 1, 2016, and December 31, 2018. The NRD is a national, all-payer database sponsored by the Healthcare Cost and Utilization Project, a health care body established by the Agency for Healthcare Research and Quality. The NRD includes in-hospital and 1-year readmission data derived from US hospitals in 28 geographically dispersed states and is designed to represent approximately 58.7% of all US hospitalizations.^8^ The NRD uses a deidentified unique number for every patient, tracking patients and determining readmissions across hospitals within a calendar year. The present study was deemed exempt by the Indiana University institutional review board because the database contained deidentified data sets with prior ethical committee approval; thus, the need for informed consent was waived by the board. This study followed the reporting guidelines specified by the Strengthening the Reporting of Observational Studies in Epidemiology (STROBE) reporting guideline.^9^

### Study Population

**Figure 1.**
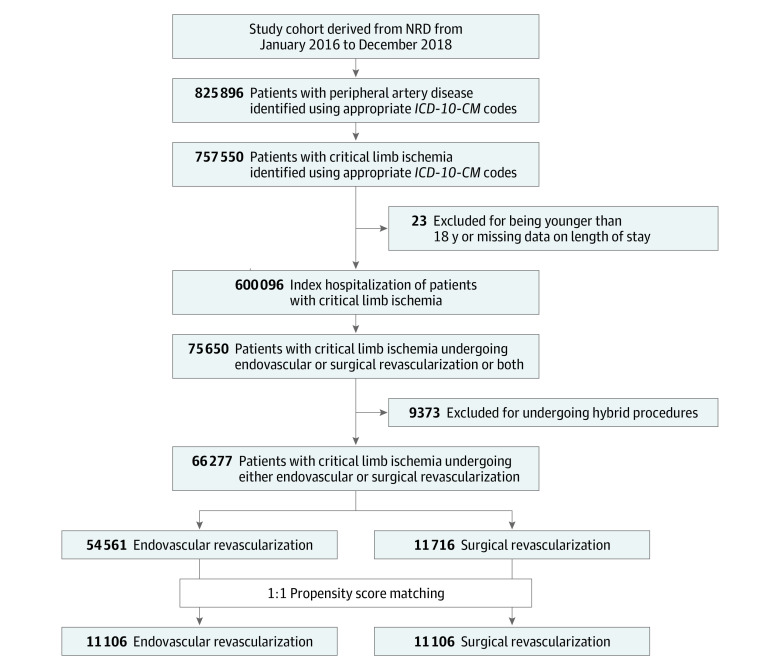
Patient Selection Flow Diagram Hybrid procedure included both endovascular revascularization and surgical revascularization during hospitalization. *ICD-10-CM* indicates *International Statistical Classification of Diseases, Tenth Revision, Clinical Modification*; NRD, Nationwide Readmissions Database.

We queried the NRD with the *International Statistical Classification of Diseases, Tenth Revision, Clinical Modification* (*ICD-10-CM*) codes to identify all adults (n = 825 896) who had a primary or secondary peripheral artery disease diagnosis. We extracted hospitalizations with CLI (n = 757 550), using appropriate *ICD-10-CM* codes as a primary or secondary diagnosis. The codes used to identify the cohort in this study have been summarized in eTable 1 in the Supplement and used in a previous study.^3^ We excluded patients younger than 18 years and missing length of stay (LOS) and derived the index hospitalizations of patients with CLI (n = 600 096). The flow diagram for patient selection has been summarized in Figure 1.

### Patient and Hospital Characteristics

We used NRD variables for patients’ age, sex, type of admission (elective or nonelective), admission day (weekday or weekend), the primary payer, and median household income for patients’ zip code.^10^ We used the Elixhauser comorbidity variables to include hypertension, diabetes, hyperlipidemia, valvular heart disease, chronic heart failure, chronic pulmonary disease, chronic kidney disease, chronic liver disease, anemia, alcohol abuse, drug abuse, obesity, coagulopathy, cancer, fluid-electrolyte disorders, and depression.^11^ *ICD-10-CM* codes were used to define the history of nonadherence, smoking, stroke or transient ischemic attack, ischemic cardiomyopathy, carotid artery disease, percutaneous coronary intervention, coronary artery bypass graft, atrial fibrillation, and end-stage kidney disease. For clinical presentation, we applied *ICD-10-CM* codes to extract patients with rest pain, ulcer, osteomyelitis, gangrene, chronic total occlusion, diffuse atherosclerosis, nontraumatic ischemic muscle infarct, bacteremia or sepsis, history of lower extremity amputation, reduced mobility, and oxygen dependence to understand the patients’ level of severity. The *ICD-10-CM* codes used to identify these variables are summarized in eTable 3 in the Supplement. We used the NRD’s hospital variables to identify hospital size according to the number of beds, teaching status, hospital location, and procedure volume. The procedure volume at each hospital was calculated by adding all the admissions with ER or SR separately. Hospitals were divided into quartiles (first, second, third, and fourth quartile) to ensure an equal number of procedures. Hospitals in the fourth quartile were considered high-volume hospitals (median number of procedures, 52 [IQR, 43-73]), and those in the first to third quartiles were considered low-volume hospitals (median number of procedures, 16 [IQR, 9-26]).

### Intervention

We divided patients with CLIs into 2 cohorts, those who underwent ER and those who underwent SR. We excluded duplicates and patients who had both procedures (hybrid procedures; n = 9373) during the same hospitalization to arrive at the final cohort of index admissions of patients with CLI who underwent either ER or SR (N = 66 277). We applied *ICD-10-CM* procedure codes to derive ER and SR (eTable 1 in the Supplement) and types of devices and procedures used for ER (eTable 2 in the Supplement).

### Study Outcomes

The study’s primary outcome was cumulative incidence of major amputation at 6 months after hospital discharge. In-hospital amputation was not included in the outcome because it could be a part of the initial revascularization strategy. We did not use amputation-free survival for the outcome because out-of-hospital mortality is not captured by the NRD. Secondary outcomes were cumulative incidence of mortality, in-hospital safety outcome (composite of acute kidney injury, major bleeding, and vascular complication), postdischarge major adverse cardiovascular events (composite of all-cause mortality, stroke, and myocardial infarction) at 6 months, and unplanned all-cause readmission at 6 months. Appropriate *ICD-10-CM* codes were used to identify all secondary outcomes (eTable 4 in the Supplement). For mortality, 6-month outcomes were cumulatively in-hospital and postdischarge events within 6 months. For unplanned all-cause readmissions, we excluded elective readmissions. Other outcomes were the postprocedure LOS and hospital cost of the index hospitalization.

### Statistical Analysis

We presented categorical variables as numbers and percentages and continuous variables as mean (SD) values or median (IQR) values based on distribution. Categorical variables were compared between the 2 groups with the χ^2^ test or the Fisher exact test, whereas continuous variables with normal distribution were compared with the *t* test, and those not normally distributed were compared with the Wilcoxon rank sum test.

We generated propensity score–matched cohorts for patients who underwent ER or SR. The propensity score was generated with all covariates described in Table 1, including patients’ demographic characteristics, comorbid conditions, hospital characteristics, clinical presentation, admission type, admission day, primary payer, and household income through multivariable logistic regression. Patients with similar propensity scores in the 2 groups were matched with a 1-to-1 scheme without replacement, using a Greedy method. Maximum propensity score differences (caliper width) of 0.2 were permitted between matched-pair observations to keep standardized differences less than 5%. Patients without matched observations were excluded. The appropriateness of all models was assessed by the receiver operating characteristic curve C statistic, which was greater than 0.75. The standardized difference was used to assess the balance of variables between 2 matched cohorts and depicted graphically (eFigure in the Supplement). For patients who died during the index hospitalization, time to death was calculated by postprocedure LOS. For patients who died after hospital discharge, time to mortality was calculated by adding postprocedure LOS of index hospitalization, time to readmission, and LOS of readmission. In calculating 6-month unplanned all-cause readmission, we excluded patients who died in the hospital. The total hospital costs were calculated by multiplying total hospital charges by the corresponding cost to charge ratio, and it represents the total cost of resources used for the inpatient hospital stay, including the cost of the procedure. All costs were adjusted for inflation or the consumer price index.

**Table 1.** Baseline Characteristics of Patients Who Underwent ER vs SR for Critical Limb Ischemia

Characteristic	Before matching, No. (%)			After matching, No. (%)		
	ER (n = 54 456)	SR (n = 11 716)	*P* value	ER (n = 11 106)	SR (n = 11 106)	SMD
Age, mean (SD), y	69.4 (12)	68.6 (11)	<.001	68.6 (12)	68.7 (11.1)	1.4
Sex						
Male	34 036 (62.5)	7652 (65.3)	<.001	7225 (65.1)	7242 (65.2)	0.3
Female	20 420 (37.5)	4038 (34.5)		3881 (34.9)	3864 (34.8)	
Comorbidities						
History of nonadherence	3663 (6.7)	505 (4.3)	<.001	476 (4.3)	480 (4.3)	0.2
Hypertension	47 677 (87.6)	9995 (85.3)	<.001	9509 (85.6)	9500 (85.5)	0.2
Diabetes	40 846 (75.0)	7547 (64.4)	<.001	7075 (63.7)	7184 (64.7)	2.1
Hyperlipidemia	30 849 (56.6)	7180 (61.3)	<.001	6887 (62.0)	6793 (61.2)	1.7
History of stroke or TIA	6066 (11.1)	1286 (11.0)	.66	1217 (11.0)	1235 (11.1)	0.5
Ischemic cardiomyopathy	25 687 (47.2)	5567 (47.5)	.35	5352 (48.2)	5285 (47.6)	1.2
Carotid artery disease	1528 (2.8)	444 (3.8)	<.001	446 (4.0)	423 (3.8)	1.2
Prior PCI	5660 (10.4)	1250 (10.7)	.30	1185 (10.7)	1185 (10.7)	0
Prior CABG	8114 (14.9)	1849 (15.8)	.01	1821 (16.4)	1775 (16.0)	1.1
Valvular heart disease	5194 (9.5)	1039 (8.9)	.03	958 (8.6)	982 (8.8)	0.8
Chronic heart failure	18 268 (33.5)	3109 (26.5)	<.001	2981 (26.8)	2961 (26.7)	0.4
Atrial fibrillation	7593 (13.9)	1339 (11.4)	<.001	1256 (11.3)	1281 (11.5)	0.7
Chronic pulmonary disease	11 943 (21.9)	3034 (25.9)	<.001	2907 (26.2)	2842 (25.6)	1.4
Chronic kidney disease	24 867 (45.7)	3852 (32.9)	<.001	3897 (35.1)	3678 (33.1)	4.1
End-stage kidney disease	9268 (17.0)	958 (8.2)	<.001	820 (7.4)	912 (8.2)	2.5
Chronic liver disease	1793 (3.3)	335 (2.9)	.02	338 (3.0)	321 (2.9)	0.9
Anemia	3657 (6.7)	645 (5.5)	<.001	613 (5.5)	608 (5.5)	0.2
Smoker	16 327 (30.0)	4225 (36.1)	<.001	4083 (36.8)	3972 (35.8)	2.1
Alcohol abuse	2132 (3.9)	581 (5.0)	<.001	560 (5.0)	540 (4.9)	0.9
Drug abuse	1627 (3.0)	363 (3.1)	.50	353 (3.2)	337 (3.0)	0.8
Obesity	9010 (16.5)	1803 (15.4)	.003	1673 (15.1)	1716 (15.5)	1.1
Coagulopathy	3038 (5.6)	626 (5.3)	.33	594 (5.3)	593 (5.3)	0
Cancer	1571 (2.9)	280 (2.4)	.05	227 (2.0)	226 (2.0)	0.1
Fluid electrolyte imbalance	18 806 (34.5)	3307 (28.2)	<.001	3140 (28.3)	3145 (28.3)	0.1
Depression	6123 (11.2)	1319 (11.3)	.91	1281 (11.5)	1240 (11.2)	1.2
Elixhauser Comorbidity Index, median (IQR)	5 (4-6)	5 (3-6)	<.001	5 (3-6)	5 (3-6)	0.2
Clinical presentation						
Rest pain	2094 (3.8)	800 (6.8)	<.001	762 (6.9)	734 (6.6)	1.1
Ulcer	12 257 (22.5)	3523 (30.1)	<.001	3395 (30.6)	3333 (30.0)	1.3
Osteomyelitis	14 109 (25.9)	1790 (15.3)	<.001	1590 (14.3)	1702 (15.3)	2.5
Gangrene	16 989 (31.2)	3546 (30.3)	.06	3541 (31.9)	3377 (30.4)	3.2
Chronic total occlusion	7696 (14.1)	1241 (10.6)	<.001	1188 (10.7)	1201 (10.8)	0.4
Diffuse atherosclerosis	1386 (2.5)	386 (3.3)	<.001	401 (3.6)	365 (3.3)	1.9
Nontraumatic ischemic muscle infarct	74 (0.1)	15 (0.1)	.84	19 (0.2)	15 (0.1)	0.4
Bacteremia/sepsis	7988 (14.7)	976 (8.3)	<.001	894 (8.0)	933 (8.4)	1.1
Prior lower extremity amputation	9428 (17.3)	1736 (14.8)	<.001	1619 (14.6)	1590 (14.3)	0.7
Reduced mobility	2100 (3.9)	377 (3.2)	.001	333 (3.0)	346 (3.1)	0.6
Oxygen dependent	1098 (2.0)	211 (1.8)	.14	203 (1.8)	205 (1.8)	0.1
Endovascular devices and procedures						
Drug-coated balloon	15 988 (29.4)	NA	NA	3053 (27.5)	NA	NA
Drug-coated balloon plus drug-eluting stent	2603 (4.8)	NA	NA	517 (4.7)	NA	NA
Drug-coated balloon plus bare-metal stent	2960 (5.4)	NA	NA	635 (5.7)	NA	NA
Uncoated percutaneous transluminal angioplasty balloon	52 373 (96.2)	NA	NA	9706 (87.4)	NA	NA
Drug-eluting stent	8662 (15.9)	NA	NA	1891 (17.0)	NA	NA
Bare-metal stent	16 968 (31.2)	NA	NA	3731 (33.6)	NA	NA
Atherectomy	29 305 (53.8)	NA	NA	5892 (53.1)	NA	NA
Hospital bed size^a^						
Small	6088 (11.2)	1303 (11.1)	<.001	1158 (10.4)	1246 (11.2)	1.3
Medium	15 798 (29.0)	3108 (26.5)		3048 (27.4)	2968 (26.7)	
Large	32 570 (59.8)	7279 (62.1)		6900 (62.1)	6892 (62.1)	
Hospital teaching status^b^						
Nonteaching	15 233 (28.0)	2474 (21.1)	<.001	2231 (20.1)	2375 (21.4)	3.0
Teaching	39 223 (72.0)	9216 (78.7)		8875 (79.9)	8731 (78.6)	
Hospital location^c^						
Nonurban	1795 (3.3)	361 (3.1)	.23	326 (2.9)	354 (3.2)	1.4
Urban	51 140 (93.9)	11 042 (94.2)		10 780 (97.1)	10 752 (96.8)	
Hospitals stratified by annual procedure volume, quartile^d^						
First	14 219 (26.1)	3084 (26.3)	<.001	2767 (24.9)	3006 (27.1)	2.9
Second	12 523 (23.0)	2989 (25.5)		2504 (22.5)	2911 (26.2)	
Third	13 001 (23.9)	2552 (21.8)		2871 (25.9)	2489 (22.4)	
Fourth	13 192 (24.2)	2778 (23.7)		2964 (26.7)	2700 (24.3)	
Type of admission^e^						
Nonelective	44 266 (81.3)	5875 (50.1)	<.001	5401 (48.6)	5647 (50.8)	4.9
Elective	10 190 (18.7)	5815 (49.6)		5705 (51.4)	5459 (49.2)	
Admission day						
Weekday	46 328 (85.1)	10 644 (90.9)	<.001	10 143 (91.3)	10 093 (90.9)	1.4
Weekend	8128 (14.9)	1046 (8.9)		963 (8.7)	1013 (9.1)	
Primary payer^f^						
Medicare	40 149 (73.7)	8398 (71.7)	<.001	7998 (72.0)	7994 (72.0)	0.5
Medicaid	5525 (10.1)	1188 (10.1)		1151 (10.4)	1103 (9.9)	
Private insurance	6558 (12.0)	1745 (14.9)		1496 (13.5)	1648 (14.8)	
Median household income, percentile^g^						
0-25	18 056 (33.2)	3411 (29.1)	<.001	3522 (31.7)	3291 (29.6)	2.7
26-50	14 692 (27.0)	3297 (28.1)		2793 (25.1)	3170 (28.5)	
51-75	12 349 (22.7)	2849 (24.3)		2780 (25.0)	2729 (24.6)	
76-100	8691 (16.0)	2000 (17.1)		2011 (18.1)	1916 (17.3)	

Abbreviations: CABG, coronary artery bypass graft; ER, endovascular revascularization; NA, not applicable; PCI, percutaneous coronary intervention; SMD, standardized mean difference; SR, surgical revascularization; TIA, transient ischemic attack.

^a^ The bed-size cutoff points, divided into small, medium, and large, were selected so that approximately one-third of the hospitals in a given region, location, and teaching status combination would fall within each bed-size category.

^b^ A hospital is considered to be a teaching hospital if it has an American Medical Association–approved residency program.

^c^ Urban-rural designation of the hospital according to the county of the hospital as identified by the American Hospital Association. The 4-level categorization is a simplified adaptation of the urban influence codes (UICs). The 12 categories of the UICs are combined into 4 broader categories that differentiate between large and small metropolitan, micropolitan, and a nonurban residual.

^d^ First to third quartiles were considered low-volume hospitals and fourth quartile was considered high-volume hospitals. Median annual procedure volume (IQR) in each quartile: first quartile, 8 (4-12); second quartile, 19 (16-22); third quartile, 30 (27-35); and fourth quartile, 52 (43-73).

^e^ Type of admission indicates whether the admission to the hospital was elective or not.

^f^ Medicare includes both fee-for-service and managed care Medicare patients. Medicaid includes both fee-for-service and managed care Medicaid patients. Private insurance includes Blue Cross, commercial carriers, and private health maintenance organizations and preferred provider organizations.

^g^ Represents a quartile classification of the estimated median household income of residents within the patient’s zip code.

We conducted logistic regression for safety outcomes to compute the odds ratio with 95% CI in the matched cohort. Time-to-event analyses were conducted with Kaplan-Meier curves, and Kaplan-Meier estimates were generated for major amputation, all-cause mortality, and unplanned all-cause readmission (eTable 5 in the Supplement). We conducted Cox proportional hazards regression for major amputation, mortality, and unplanned all-cause readmission to compute the hazard ratio with 95% CI. For major amputation, we applied competing risk regression analysis to take concurrent mortality into account. The proportionality assumption was not violated for the Cox model for these outcomes, with the global test *P* > .05. For LOS and cost, we applied a generalized linear model with gamma family and log link to compute coefficients with 95% CI.

A subgroup analysis was performed for major amputation to look for sources of heterogeneity. Clinically important subgroups included age, sex, hypertension, diabetes, smoking, gangrene, osteomyelitis, chronic kidney disease, and low- and high-volume hospitals. We did not include rest pain as a subgroup because of the small sample in the included cohort; only 2094 of 54 456 patients (3.8%) in the ER group and 800 of 11 716 (6.8%) in the SR group had rest pain before intervention. We created a spline graph comparing ER with SR for a 6-month major amputation by hospital procedure volume.

### Unmeasured Bias Analysis

We conducted previously validated falsification end point and E-value analysis to evaluate the robustness of our findings.^12,13,14,15,16^ In the falsification method, we selected an alternative outcome that may not be expected to be causally affected by the treatment being studied. Then, we assessed whether the study intervention (ER) was associated with alternative outcomes by using a similar method to assess other study outcomes. If no treatment effect was observed for the alternative outcome, it supported but did not prove that there may have been a causal treatment effect for the study outcomes. We chose a composite of pneumonia and gastrointestinal and urinary tract infection at readmission as an alternative outcome and studied the association with interventions (ER). The E-value identifies the minimum strength of association that unmeasured confounders need to have with both treatment and outcome, conditional on measured covariates, to fully explain the observed association. This strength of association estimates what the relative risk may have to be for any unmeasured confounder to overcome the observed association of study intervention (ER) with study outcomes.

All *P* values were 2 sided, with *P* < .05 considered statistically significant. All statistical analyses were conducted with unweighted samples with Stata, version 16.1 (StataCorp). Data analyses were conducted from January 1, 2022, to February 28, 2022.

## Results

### Description of Study Population

A total of 66 277 patients were identified between 2016 and 2018 who underwent ER or SR for CLI. The mean (SD) age of the cohort was 69.3 (12) years, 34 036 patients were male (62.5%), and 20 420 were female (37.5%). The NRD does not provide racial and ethnic categories. Of the 66 277 patients identified, 54 546 (82.3%) underwent ER and 11 731 (17.7%) underwent SR. After propensity score matching, 11 106 matched pairs were found with a median follow-up of 180 days (IQR, 90-270 days).

### Baseline Characteristics

Table 1 shows baseline characteristics of patients who underwent ER and patients who underwent SR before and after matching. Before matching, the ER population was older and had more female patients than the SR group. The ER cohort had a higher percentage of patients with a history of nonadherence, hypertension, diabetes, valvular heart disease, heart failure, atrial fibrillation, chronic kidney disease, end-stage kidney disease, chronic liver disease, anemia, obesity, cancer, and fluid electrolyte imbalance. The SR cohort had a higher percentage of patients with hyperlipidemia, carotid artery disease, prior coronary artery bypass graft, chronic pulmonary disease, smoking, and alcohol abuse.

In terms of clinical presentation, the ER cohort had a higher percentage of patients with osteomyelitis, chronic total occlusion, systemic infection (bacteremia or sepsis), history of lower extremity amputation, and reduced mobility. In contrast, the SR cohort had a higher percentage of patients with rest pain, ulcer, and diffuse atherosclerosis.

Among the 54 456 patients who underwent ER, 15 988 (29.4%) received a drug-coated balloon, 8662 (15.9%) received a drug-eluting stent, 16 968 (31.2%) received a bare-metal stent, and 29 305 (53.8%) had undergone atherectomy. Most SR and ER procedures were conducted in hospitals with large bed sizes, teaching hospitals, and urban hospital locations. For ER and SR, more admissions were nonelective and on weekdays. Both ER and SR cohorts had Medicare as the primary payer and a lower median household income per zip code.

### Outcomes

**Figure 2.**
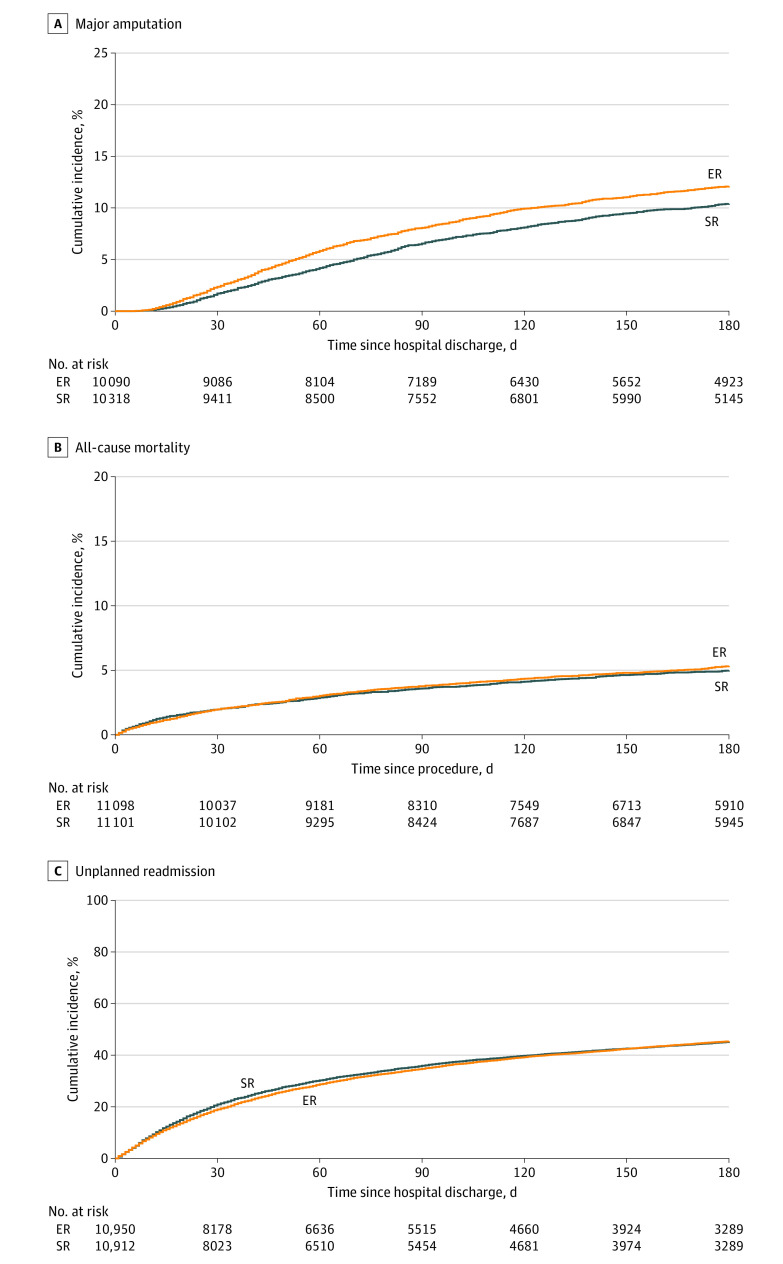
Major Amputation, All-Cause Mortality, and Unplanned Readmission for Patients Undergoing Endovascular Revascularization (ER) vs Surgical Revascularization (SR) A, Kaplan-Meier graphs showing major amputation in patients with critical limb ischemia. Compared with SR, ER had a slightly higher risk of major amputation at 6 months (997 of 10 090 [9.9%] vs 869 of 10 318 [8.4%]; hazard ratio [HR], 1.18; 95% CI, 1.08-1.29; *P* = .001). The HR is calculated from competing risk regression analysis. B, Kaplan-Meier graphs showing mortality among patients with critical limb ischemia. Compared with SR, ER had similar all-cause mortality at 6 months (517 [4.7%] vs 490 [4.4%]; HR, 1.06; 95% CI, 0.93-1.20; *P* = .39). C, Kaplan-Meier graphs showing unplanned all-cause readmission among patients with critical limb ischemia. Risk of unplanned readmission at 6 months was similar for ER and SR (4278 of 10 950 [39.1%] vs 4313 of 10 912 [39.5%]; HR, 0.98; 95% CI, 0.94-1.02; *P* = .39).

Endovascular revascularization was associated with an 18% higher risk of amputation compared with SR at 6 months (997 of 10 090 [9.9%] vs 869 of 10 318 [8.4%]; hazard ratio, 1.18; 95% CI, 1.08-1.29; *P* = .001) (Table 2; Figure 2A).

**Table 2.** Outcomes With ER vs SR in a Propensity Score–Matched Cohort of Patients With Critical Limb Ischemia

Outcome	ER (n = 11 106)	SR (n = 11 106)
**Primary outcome: major amputation**		
No. of patients with events	997	869
Cumulative event rate, %^a^		
At 30 d	2.4	1.7
At 3 mo	8.1	6.5
At 6 mo	12.1	10.4
HR (95% CI)^b^	1.18 (1.08-1.29)	
*P* value	.001	
E-value, point estimate (lower limit of the 95% CI)	1.6 (1.4)	
**Mortality^c^**		
No. of patients with events	517	490
Cumulative event rate, %^a^		
In-hospital	1.4	1.8
At 30 d	2.4	2.5
At 3 mo	3.8	3.6
At 6 mo	5.5	5.1
HR (95% CI)	1.06 (0.93-1.20)	
*P* value	.39	
**Safety (AKI, major bleeding, vascular complication)**		
No. of patients with events	2584	2979
In-hospital cumulative event rate, %	23.3	26.8
OR (95% CI)	0.83 (0.78-0.88)	
*P* value	<.001	
E-value, point estimate (lower limit of the 95% CI)	1.7 (1.5)	
**Major adverse cardiovascular event (MI, stroke, death)**		
No. of patients with events	637	635
Cumulative event rate, %^a^		
At 30 d	4.0	4.4
At 3 mo	4.9	5.2
At 6 mo	5.7	5.6
HR (95% CI)	1.00 (0.90-1.12)	
*P* value	.95	
**All-cause readmission (unplanned)**		
No. of patients with events	4278	4313
Cumulative event rate, %^a^		
At 30 d	19.0	21.0
At 3 mo	34.7	35.9
At 6 mo	45.4	45.1
HR (95% CI)	0.98 (0.94-1.02)	
*P* value	.39	
**Length of stay**		
Median (IQR), d	3 (2-7)	6 (3-9)
OR (95% CI)	0.75 (0.73-0.78)	
*P* value	<.001	
E-value, point estimate (lower limit of the 95% CI)	2.0 (1.8)	
**Cost**		
Median (IQR), $	27 904 (18 379-41 936)	30 942 (19 654-47 435)
OR (95% CI)	0.90 (0.88-0.92)	
*P* value	<.001	
E-value, point estimate (lower limit of the 95% CI)	1.5 (1.4)	
**Falsification end point^d^**		
At 6 mo, No. (%)	110 (1)	130 (1.2)
OR (95% CI)	0.84 (0.65-1.10)	
*P* value	.19	

Abbreviations: AKI, acute kidney injury; ER, endovascular revascularization; HR, hazard ratio; MI, myocardial infarction; OR, odds ratio; SR, surgical revascularization.

^a^ Calculated with Kaplan-Meier curve time-to-event analysis.

^b^ Calculated from competing risk regression analysis.

^c^ Indicates mortality during readmission, and out-of-hospital mortality is not captured.

^d^ Falsification end point: composite of pneumonia and gastrointestinal and urinary tract infection readmission.

 Endovascular revascularization and SR had similar all-cause mortality at 6 months (517 of 11 106 [4.7%] vs 490 [4.4%]; hazard ratio, 1.06; 95% CI, 0.93-1.20; *P* = .39) (Table 2; Figure 2B).

 The ER group had better in-hospital safety outcomes (17% less risk of composite of acute kidney injury, major bleeding, or vascular complication) compared with the SR group (2584 of 11 106 [23.3%] vs 2979 [26.8%]; odds ratio, 0.83; 95% CI, 0.78-0.88; *P* < .001) (Table 2).

Compared with the SR group at 6 months, the ER group had a similar risk of major adverse cardiovascular events (637 of 11 106 [5.7%] vs 635 of 11 106 [5.7%]; hazard ratio, 1.00; 95% CI, 0.90-1.12; *P* = .95) and unplanned all-cause readmission (4278 of 10 950 [39.1%] vs 4313 of 10 912 [39.5%]; hazard ratio, 0.98; 95% CI, 0.94-1.02; *P* = .39) (Table 2; Figure 2C).

Regarding resource use, ER was associated with 25% shorter LOS (odds ratio, 0.75; 95% CI, 0.73-0.78; *P* < .001) and 10% lower hospitalization cost (odds ratio, 0.90; 95% CI, 0.88-0.92; *P* < .001) compared with SR (Table 2).

### Unmeasured Bias Analysis: Falsification End Point and E-value Analysis

**Figure 3.**
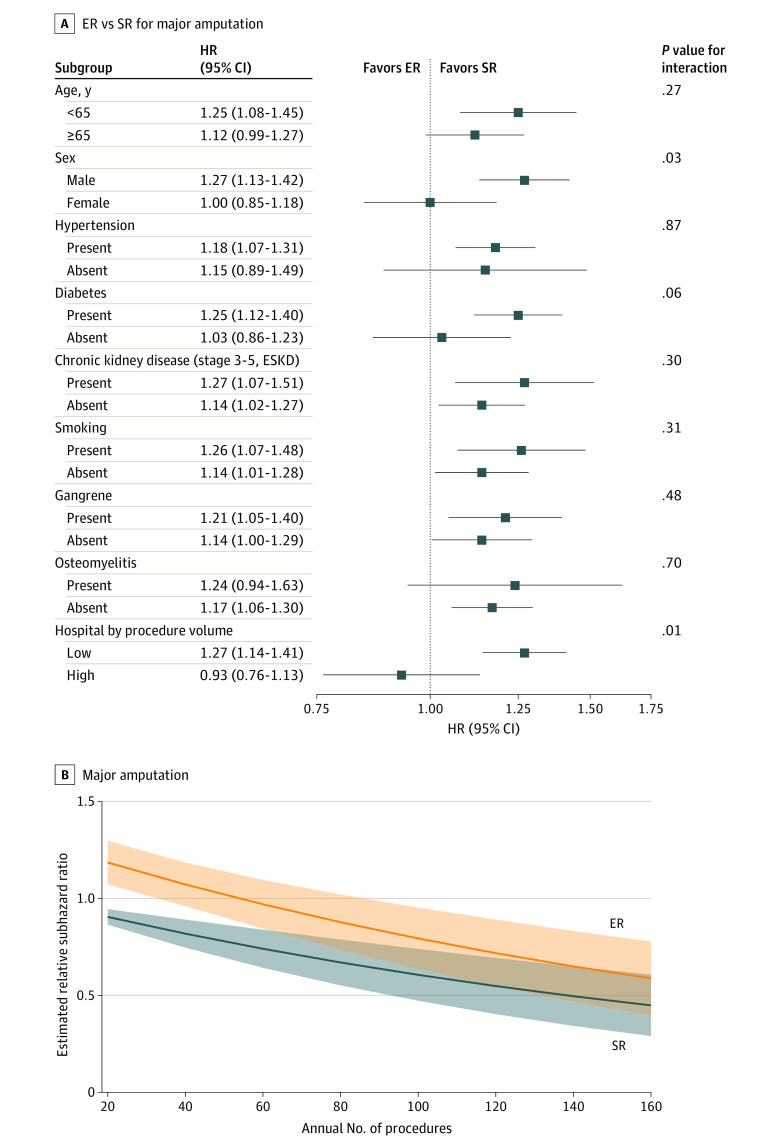
Subgroup Analysis for Major Amputation Among Patients With Critical Limb Ischemia A, Forest plot comparing ER and SR for major amputation in different subgroups. Sex, hospital procedure volume, and diabetes showed interaction with ER. B, Spline graph for major amputation comparing ER and SR by annual procedure volume (*P* = .01 for interaction). The difference in risk of major amputation between the 2 procedures decreased as the procedure volume of a hospital increased. ER indicates endovascular revascularization; ESKD, end-stage kidney disease; HR, hazard ratio; and SR, surgical revascularization.

The falsification end point was similar between the 2 groups at 6 months, supporting well-balanced groups for unmeasured confounders (odds ratio, 0.84; 95% CI, 0.65-1.10; *P* = .19). For major amputation, E-value analysis showed that unmeasured confounders required a risk ratio of 1.6 (lower limit of the 95% CI, 1.4) with treatment and amputation to explain the effect. For in-hospital safety outcomes, E-value analysis showed that unmeasured confounders require a risk ratio of 1.7 (lower limit of the 95% CI, 1.5) with treatment and amputation to explain the effect (Table 2). For the subgroup analysis, none of the proposed subgroups showed an interaction except sex (*P* = .03 for interaction), diabetes (*P* = .06 for interaction), and volume of the hospital (*P* = .007 for interaction) (Figure 3).

## Discussion

In this multicenter, retrospective cohort study of patients with CLI that compared ER with SR, we derived 4 important findings; compared with SR, ER was associated with (1) a higher risk of major amputations at 6 months, but this risk was similar when revascularization was performed in high-volume centers; (2) similar mortality, major adverse cardiovascular events, and unplanned all-cause readmission at 6 months; (3) better safety outcomes, including a lower risk of acute kidney injury, major bleeding, and vascular complications; and (4) lower resource use (shorter LOS and lower cost).

Although it is well known that CLI causes significant health care burden and mortality, to our knowledge, recent data on CLI peripheral interventions and outcomes are lacking. However, for the past 2 decades, the trend of SR has decreased with an increase in ER procedures.^3,17,18^ The Inter-Society Consensus for the Management of Peripheral Arterial Disease, along with the most recent guidelines from the American College of Cardiology and the American Heart Association, recommend revascularization as the optimal treatment.^6,19^ However, there is still debate between surgical and endovascular approaches as the first choice, given the lack of contemporary comparative data and the mixed results of observational studies.

We found an association between increased risk of major amputation and the ER group compared with the SR group at 6 months, which differs from the results of the BASIL trial, the Registry of First-Line Treatments in Patients With Critical Limb Ischemia (CRITISCH), the California state database, and the Surgical Reconstruction Versus Peripheral Intervention in Patients With Critical Limb Ischemia (SPINACH) study.^7,20,21,22^ The BASIL trial, CRITISCH, and the SPINACH study showed similar amputation-free survival rates between the 2 groups, whereas the California database showed better amputation-free survival in the ER group. The BASIL trial included few patients with acute limb ischemia, and the SPINACH study had a higher percentage of patients (55%) undergoing dialysis. Moreover, we studied major amputation rather than amputation-free survival because the NRD does not capture out-of-hospital mortality, as already mentioned. The sample sizes of the BASIL trial, CRITISCH, and the SPINACH study were smaller than ours and applied several exclusion criteria to select the final cohort, whereas this study included a patient population without stringent selection criteria. Moreover, Menard et al^23^ reported in 2016 that endovascular treatment in the BASIL trial was limited to percutaneous transluminal angioplasty alone, as was standard practice in the United Kingdom where the trial was undertaken, and the applicability of the trial result has been questioned regarding practice in the United States, which typically includes the use of stents, atherectomy, and drug-coated balloons. In accordance with the types of ER devices and procedures used in our study (Table 1) and the years of our database, our study included advanced ER. Thus, differences in patient population, duration of studies, ER techniques, and nature of outcome from our study might be the reason for the observed difference. The California state database included nonfederal California hospitals, limiting its generalizability. In addition, the California state database incorporated a few variables, such as age, sex, race and ethnicity, insurance, coronary artery disease, and kidney failure in propensity score matching. In contrast, we included more variables in propensity score matching that were associataed with CLI outcomes directly, giving more robust results for all comers than the prespecified cohort with profuse exclusion criteria (eTable 6 in the Supplement). The reason for the higher level of major amputation associated with ER is not clear; however, previous studies have shown that major tissue loss and severe infection are well-known risk factors for delayed wound healing and major amputation. It often requires abundant blood flow for limb salvage, and SR is better at restoring sufficient blood flow supply to tissue.^24,25,26^ Moreover, the subgroup analysis showed a significant interaction for sex and the volume of hospitals. In the high-volume centers, the risk of major amputation was similar between ER and SR, which could be explained by improved operator experience. Studies showed that in men, multiple sites of the arterial tree are affected compared with those in women, which could make ER less effective in men.^27,28^ Moreover, women have a higher incidence of limb-related complications after SR, which could be the reason for comparable major amputation rates between the 2 procedures among women.^29^

In this study, there was no difference in mortality between ER and SR groups at 6 months, which aligns with the results of the BASIL trial,^7^ showing similar 12-month all-cause mortality between the 2 groups. However, it remains unclear whether the evidence presented by the randomized clinical trial would remain valid in current clinical practice with the availability of newer endovascular devices and revascularization techniques. For 6-month outcomes, our result is also consistent with recently published CRITISCH^20^ results and the California state database,^21^ showing no difference in mortality.

The risk of major adverse cardiovascular events was similar among both groups in our study, which was also observed in the prospective multicenter observational SPINACH study conducted in Japan.^22^ However, the SPINACH study included only 548 Japanese patients, and there was a higher prevalence of patients undergoing dialysis (55%), who typically have extensive calcification and severe peripheral artery disease compared with those without advanced kidney disease. Moreover, novel endovascular devices, including drug-coated balloons and atherectomy devices, were not used. The 30-day unplanned readmission rate between the 2 groups was similar in our analysis, whereas the overall rate of major bleeding, vascular complications, or acute kidney injury was lower in the ER group, which is similar to the results of the 2013-2014 NRD data analysis using *ICD-9-CM* codes.^5^ Moreover, the higher cost of SR hospitalization compared with ER hospitalization can be explained by longer LOS and a higher rate of in-hospital complications.

### Strengths and Limitations

Our study needs to be interpreted in light of the following limitations. First, the NRD is an administrative database that carries the potential for miscoding. However, the Agency for Healthcare Research and Quality has quality control measures to secure best coding practices, to ensure that linkage to state-level data is verified and reliable, and to establish internal validation of diagnosis codes through multiple audits. We assumed that the initial peripheral artery disease diagnosis may have been miscoded or that the severity was not enough for patients to be considered for ER or SR because only 10% of the cohort underwent such procedures and were included in the analysis. However, ER, SR, and amputation procedures are less likely to be miscoded. Second, we did not have anatomic data, including lesion length, lesion complexity, types of bypasses used (such as the level of bypass and conduit used), and severity of the disease at presentation (ie, Rutherford or Fontaine classification), so we could not stratify results based on disease severity. However, we did show clinical presentation based on coding, and subgroup analyses were conducted for gangrene and osteomyelitis. We also showed types of ER devices and procedures (Table 1) based on coding; however, we did not have specific codes for laser atherectomy. Third, we could not calculate the Project of Ex Vivo Vein Graft Engineering via Transfection risk score, which can help to estimate the risk of amputation-free survival after CLI. Fourth, we could not assess outcomes such as a major adverse limb event, which includes major amputation and major target limb reintervention, because reintervention could occur in a different limb, which was not possible to discern from the target limb. Fifth, the NRD does not capture out-of-hospital death; hence, postdischarge mortality includes death during readmission. This limitation applies to both cohorts; thus, the calculation of the ratio would not be affected. Sixth, patients treated in an office-based outpatient center were not captured. Despite these limitations, this analysis provides valuable data on 6-month outcomes for patients with CLI from the most current, large, all-payer, nationally available database, and the multi-institutional sample makes our results generalizable. Application of falsification outcome and E-value analysis significantly decreased the possibility of selection bias and change of result due to unmeasured confounders.

## Conclusions

In a propensity score–matched cohort of patients with CLI from the NRD, ER was associated with better safety without any difference in mortality but was also associated with an increased risk of major amputation compared with SR. However, the risk of major amputation was similar when both procedures were performed at high-volume centers. Hence, the procedure selection for CLI management should be a shared and informed decision, with the patient understanding all the risks and benefits as discussed in this study.

## Notes

Supplement. **eTable 1.** *ICD-10* Codes of Variables Used for Cohort Selection **eTable 2.** *ICD-10* Procedure Codes of Devices and Procedures Used for Endovascular Revascularization **eTable 3.** *ICD-10* Codes of Variables Used for Clinical Presentation and Comorbidities **eTable 4.** *ICD-10* Codes for Outcomes **eTable 5.** Details About Time-to-Event Analysis **eTable 6.** Studies Comparing Endovascular vs Surgical Revascularization in Patients With Critical Limb Ischemia **eFigure.** Balance of Covariates Between 2 Revascularization Strategies
