# Supplementary material for: Comparison of 6-Month Outcomes of Endovascular vs Surgical Revascularization for Patients With Critical Limb Ischemia
Source: JAMA Netw Open. 2022 Aug 19;5(8):e2227746. doi: 10.1001/jamanetworkopen.2022.27746 (PMC9391961; doi:10.1001/jamanetworkopen.2022.27746)
Supplement: Supplement. — eTable 1. ICD-10 Codes of Variables Used for Cohort Selection eTable 2. ICD-10 Procedure Codes of Devices and Procedures Used for Endovascular Revascularization eTable 3. ICD-10 Codes of Variables Used for Clinical Presentation and Comorbidities eTable 4. ICD-10 Codes for Outcomes eTable 5. Details About Time-to-Event Analysis eTable 6. Studies Comparing Endovascular vs Surgical Revascularization in Patients With Critical Limb Ischemia eFigure. Balance of Covariates Between 2 Revascularization Strategies [file jamanetwopen-e2227746-s001.pdf]

Supplementary Online Content

Majmundar M, Patel KN, Doshi R, et al. Comparison of 6-month outcomes of endovascular vs surgical revascularization for patients with critical limb ischemia. *JAMA Netw Open*. 2022;5(8):e2227746. doi:10.1001/jamanetworkopen.2022.27746

eTable 1. *ICD-10* Codes of Variables Used for Cohort Selection

eTable 2. *ICD-10* Procedure Codes of Devices and Procedures Used for Endovascular Revascularization

eTable 3. *ICD-10* Codes of Variables Used for Clinical Presentation and Comorbidities

eTable 4. *ICD-10* Codes for Outcomes

eTable 5. Details About Time-to-Event Analysis

eTable 6. Studies Comparing Endovascular vs Surgical Revascularization in Patients With Critical Limb Ischemia

eFigure. Balance of Covariates Between 2 Revascularization Strategies

This supplementary material has been provided by the authors to give readers additional information about their work.

© 2022 Majmundar M et al. *JAMA Network Open.*

eTable 1. *ICD-10* Codes of Variables Used for Cohort Selection

Peripheral Artery Disease
I700, I70201, I70202, I70203, I70208, I70209, I70211, I70212, I70213, I70218, I70219, I70221, I70222, I70223, I70228, I70229, I70231, I70232, I70233, I70234, I70235, I70238, I70239, I70241, I70242, I70243, I70244, I70245, I70248, I70249, I7025, I70261, I70262, I70263, I70268, I70269, I70291, I70292, I70293, I70298, I70299, I70301, I70302, I70303, I70308, I70309, I70311, I70312, I70313, I70318, I70319, I70321, I70322, I70323, I70328, I70329, I70331, I70332, I70333, I70334, I70335, I70338, I70339, I70341, I70342, I70343, I70344, I70345, I70348, I70349, I7035, I70361, I70362, I70363, I70368, I70369, I70391, I70392, I70393, I70398, I70399, I70401, I70402, I70403, I70408, I70409, I70411, I70412, I70413, I70418, I70419, I70421, I70422, I70423, I70428, I70429, I70431, I70432, I70433, I70434, I70435, I70438, I70439, I70441, I70442, I70443, I70444, I70445, I70448, I70449, I7045, I70461, I70462, I70463, I70468, I70469, I70491, I70492, I70493, I70498, I70499, I70501, I70502, I70503, I70508, I70509, I70511, I70512, I70513, I70518, I70519, I70521, I70522, I70523, I70528, I70529, I70531, I70532, I70533, I70534, I70535, I70538, I70539, I70541, I70542, I70543, I70544, I70545, I70548, I70549, I7055, I70561, I70562, I70563, I70568, I70569, I70591, I70592, I70593, I70598, I70599, I70601, I70602, I70603, I70608, I70609, I70611, I70612, I70613, I70618, I70619, I70621, I70622, I70623, I70628, I70629, I70631, I70632, I70633, I70634, I70635, I70638, I70639, I70641, I70642, I70643, I70644, I70645, I70648, I70649, I7065, I70661, I70662, I70663, I70668, I70669, I70691, I70692, I70693, I70698, I70699, I70701, I70702, I70703, I70708, I70709, I70711, I70712, I70713, I70718, I70719, I70721, I70722, I70723, I70728, I70729, I70731, I70732, I70733, I70734, I70735, I70738, I70739, I70741, I70742, I70743, I70744, I70745, I70748, I70749, I7075, I70761, I70762, I70763, I70768, I70769, I70791, I70792, I70793, I70798, I70799, I7090, I7091, I7092, I798, I739, I731, I743, I745, I96, E0851, E0852, E0951, E0952, E1051, E1052, E1151, E1152, E1165

© 2022 Majmundar M et al. *JAMA Network Open.*

**Critical Limb Ischemia**
L971, L9710, L97101, L97102, L97103, L97104, L97105, L97106, L97108, L97109, L9711, L97111, L97112, L97113, L97114, L97115, L97116, L97118, L97119, L9712, L97121, L97122, L97123, L97124, L97125, L97126, L97128, L97129, L972, L9720, L97201, L97202, L97203, L97204, L97205, L97206, L97208, L97209, L9721, L97211, L97212, L97213, L97214, L97215, L97216, L97218, L97219, L9722, L97221, L97222, L97223, L97224, L97225, L97226, L97228, L97229, L973, L9730, L97301, L97302, L97303, L97304, L97305, L97306, L97308, L97309, L9731, L97311, L97312, L97313, L97314, L97315, L97316, L97318, L97319, L9732, L97321, L97322, L97323, L97324, L97325, L97326, L97328, L97329, L974, L9740, L97401, L97402, L97403, L97404, L97405, L97406, L97408, L97409, L9741, L97411, L97412, L97413, L97414, L97415, L97416, L97418, L97419, L9742, L97421, L97422, L97423, L97424, L97425, L97426, L97428, L97429, L975, L9750, L97501, L97502, L97503, L97504, L97505, L97506, L97508, L97509, L9751, L97511, L97512, L97513, L97514, L97515, L97516, L97518, L97519, L9752, L97521, L97522, L97523, L97524, L97525, L97526, L97528, L97529, L978, L9780, L97801, L97802, L97803, L97804, L97805, L97806, L97808, L97809, L9781, L97811, L97812, L97813, L97814, L97815, L97816, L97818, L97819, L9782, L97821, L97822, L97823, L97824, L97825, L97826, L97828, L97829, L979, L9790, L97901, L97902, L97903, L97904, L97905, L97906, L97908, L97909, L9791, L97911, L97912, L97913, L97914, L97915, L97916, L97918, L97919, L9792, L97921, L97922, L97923, L97924, L97925, L97926, L97928, L97929, M8625, M86251, M86252, M86259, M8626, M86261, M86262, M86269, M8627, M86271, M86272, M86279, M8615, M86151, M86152, M86159, M8616, M86161, M86162, M86169, M8617, M86171, M86172, M86179, M8665, M86651, M86652, M86659, M8666, M86661, M86662, M86669, M8667, M86671, M86672, M86679, L0303, L03031, L03032, L03039, L03115, L03116, L03119

© 2022 Majmundar M et al. *JAMA Network Open.*

Surgical Revascularization

© 2022 Majmundar M et al. *JAMA Network Open.*

0410096, 0410097, 0410098, 0410099, 041009B, 041009C, 041009D, 041009F, 041009G, 041009H, 041009J, 041009K, 041009Q, 041009R, 04100A6, 04100A7, 04100A8, 04100A9, 04100AB, 04100AC, 04100AD, 04100AF, 04100AG, 04100AH, 04100AJ, 04100AK, 04100AQ, 04100AR, 04100J6, 04100J7, 04100J8, 04100J9, 04100JB, 04100JC, 04100JD, 04100JF, 04100JG, 04100JH, 04100JJ, 04100JK, 04100JQ, 04100JR, 04100K6, 04100K7, 04100K8, 04100K9, 04100KB, 04100KC, 04100KD, 04100KF, 04100KG, 04100KH, 04100KJ, 04100KK, 04100KQ, 04100KR, 04100Z6, 04100Z7, 04100Z8, 04100Z9, 04100ZB, 04100ZC, 04100ZD, 04100ZF, 04100ZG, 04100ZH, 04100ZJ, 04100ZK, 04100ZQ, 04100ZR, 0410496, 0410497, 0410498, 0410499, 041049B, 041049C, 041049D, 041049F, 041049G, 041049H, 041049J, 041049K, 041049Q, 041049R, 04104A6, 04104A7, 04104A8, 04104A9, 04104AB, 04104AC, 04104AD, 04104AF, 04104AG, 04104AH, 04104AJ, 04104AK, 04104AQ, 04104AR, 04104J6, 04104J7, 04104J8, 04104J9, 04104JB, 04104JC, 04104JD, 04104JF, 04104JG, 04104JH, 04104JJ, 04104JK, 04104JQ, 04104JR, 04104K6, 04104K7, 04104K8, 04104K9, 04104KB, 04104KC, 04104KD, 04104KF, 04104KG, 04104KH, 04104KJ, 04104KK, 04104KQ, 04104KR, 04104Z6, 04104Z7, 04104Z8, 04104Z9, 04104ZB, 04104ZC, 04104ZD, 04104ZF, 04104ZG, 04104ZH, 04104ZJ, 04104ZK, 04104ZQ, 04104ZR, 041C09H, 041C09J, 041C09K, 041C0AH, 041C0AJ, 041C0AK, 041C0JH, 041C0JJ, 041C0JK, 041C0KH, 041C0KJ, 041C0KK, 041C0ZH, 041C0ZJ, 041C0ZK, 041C49H, 041C49J, 041C49K, 041C4AH, 041C4AJ, 041C4AK, 041C4JH, 041C4JJ, 041C4JK, 041C4KH, 041C4KJ, 041C4KK, 041C4ZH, 041C4ZJ, 041C4ZK, 041D09H, 041D09J, 041D09K, 041D0AH, 041D0AJ, 041D0AK, 041D0JH, 041D0JJ, 041D0JK, 041D0KH, 041D0KJ, 041D0KK, 041D0ZH, 041D0ZJ, 041D0ZK, 041D49H, 041D49J, 041D49K, 041D4AH, 041D4AJ, 041D4AK, 041D4JH, 041D4JJ, 041D4JK, 041D4KH, 041D4KJ, 041D4KK, 041D4ZH, 041D4ZJ, 041D4ZK, 041E09H, 041E09J, 041E09K, 041E09P, 041E09Q, 041E0AH, 041E0AJ, 041E0AK, 041E0AP, 041E0AQ, 041E0JH, 041E0JJ, 041E0JK, 041E0JP, 041E0JQ, 041E0KH, 041E0KJ, 041E0KK, 041E0KP, 041E0KQ, 041E0ZH, 041E0ZJ, 041E0ZK, 041E0ZP, 041E0ZQ, 041E49H, 041E49J, 041E49K, 041E49P, 041E49Q, 041E4JH, 041E4JJ, 041E4JK, 041E4JP, 041E4JQ, 041E4KH, 041E4KJ, 041E4KK, 041E4KP, 041E4KQ, 041E4ZH, 041E4ZJ, 041E4ZK, 041E4ZP, 041E4ZQ, 041F09H, 041F09J, 041F09K, 041F09P, 041F09Q, 041F0AH, 041F0AJ, 041F0AK, 041F0AP, 041F0AQ, 041F0JH, 041F0JJ, 041F0JK, 041F0JP, 041F0JQ, 041F0KH, 041F0KJ, 041F0KK, 041F0KP, 041F0KQ, 041F0ZH, 041F0ZJ, 041F0ZK, 041F0ZP, 041F0ZQ, 041F49H, 041F49J, 041F49K, 041F49P, 041F49Q, 041F4AH, 041F4AJ, 041F4AK, 041F4AP, 041F4AQ, 041F4JH, 041F4JJ, 041F4JK, 041F4JP, 041F4JQ, 041F4KH, 041F4KJ, 041F4KK, 041F4KP, 041F4KQ, 041F4ZH, 041F4ZJ, 041F4ZK, 041F4ZP, 041F4ZQ, 041H09H, 041H09J, 041H09K, 041H09P, 041H09Q, 041H0AH, 041H0AJ, 041H0AK, 041H0AP, 041H0AQ, 041H0JH, 041H0JJ, 041H0JK, 041H0JP, 041H0JQ, 041H0KH, 041H0KJ, 041H0KK, 041H0KP, 041H0KQ, 041H0ZH, 041H0ZJ, 041H0ZK, 041H0ZP, 041H0ZQ, 041H49H, 041H49J, 041H49K, 041H49P, 041H49Q, 041H4AH, 041H4AJ, 041H4AK, 041H4AP, 041H4AQ, 041H4JH, 041H4JJ, 041H4JK, 041H4JP, 041H4JQ, 041H4KH, 041H4KJ, 041H4KK, 041H4KP, 041H4KQ, 041H4ZH, 041H4ZJ, 041H4ZK, 041H4ZP, 041H4ZQ, 041J09H, 041J09J, 041J09K, 041J09P, 041J09Q, 041J0AH, 041J0AJ, 041J0AK, 041J0AP, 041J0AQ, 041J0JH, 041J0JJ, 041J0JK, 041J0JP, 041J0JQ, 041J0KH, 041J0KJ, 041J0KK, 041J0KP, 041J0KQ, 041J0ZH, 041J0ZJ, 041J0ZK, 041J0ZP, 041J0ZQ, 041J49H, 041J49J, 041J49K, 041J49P, 041J49Q, 041J4AH, 041J4AJ, 041J4AK, 041J4AP, 041J4AQ, 041J4JH, 041J4JJ, 041J4JK, 041J4JP, 041J4JQ, 041J4KH, 041J4KJ, 041J4KK, 041J4KP, 041J4KQ, 041J4ZH, 041J4ZJ, 041J4ZK, 041J4ZP, 041J4ZQ, 0410090, 0410091, 0410092, 04100A0, 041C096, 041C097,

© 2022 Majmundar M et al. *JAMA Network Open.*

041C098, 041C099, 041C09B, 041C09C, 041C09D, 041C09F, 041C09G, 041C09H, 041C09J, 041C09K, 041C09Q, 041C09R, 041C0A6, 041C0A7, 041C0A8, 041C0A9, 041C0AB, 041C0AC, 041C0AD, 041C0AF, 041C0AG, 041C0AH, 041C0AJ, 041C0AK, 041C0AQ, 041C0AR, 041C0J6, 041C0J7, 041C0J8, 041C0J9, 041C0JB, 041C0JC, 041C0JD, 041C0JF, 041C0JG, 041C0JH, 041C0JJ, 041C0JK, 041C0JQ, 041C0JR, 041C0K6, 041C0K7, 041C0K8, 041C0K9, 041C0KB, 041C0KC, 041C0KD, 041C0KF, 041C0KG, 041C0KH, 041C0KJ, 041C0KK, 041C0KQ, 041C0KR, 041C0Z6, 041C0Z7, 041C0Z8, 041C0Z9, 041C0ZB, 041C0ZC, 041C0ZD, 041C0ZF, 041C0ZG, 041C0ZH, 041C0ZJ, 041C0ZK, 041C0ZQ, 041C0ZR, 041C496, 041C497, 041C498, 041C499, 041C49B, 041C49C, 041C49D, 041C49F, 041C49G, 041C49H, 041C49J, 041C49K, 041C49Q, 041C49R, 041C4A6, 041C4A7, 041C4A8, 041C4A9, 041C4AB, 041C4AC, 041C4AD, 041C4AF, 041C4AG, 041C4AH, 041C4AJ, 041C4AK, 041C4AQ, 041C4AR, 041C4J6, 041C4J7, 041C4J8, 041C4J9, 041C4JB, 041C4JC, 041C4JD, 041C4JF, 041C4JG, 041C4JH, 041C4JJ, 041C4JK, 041C4JQ, 041C4JR, 041C4K6, 041C4K7, 041C4K8, 041C4K9, 041C4KB, 041C4KC, 041C4KD, 041C4KF, 041C4KG, 041C4KH, 041C4KJ, 041C4KK, 041C4KQ, 041C4KR, 041C4Z6, 041C4Z7, 041C4Z8, 041C4Z9, 041C4ZB, 041C4ZC, 041C4ZD, 041C4ZF, 041C4ZG, 041C4ZH, 041C4ZJ, 041C4ZK, 041C4ZQ, 041C4ZR, 041D096, 041D097, 041D098, 041D099, 041D09B, 041D09C, 041D09D, 041D09F, 041D09G, 041D09H, 041D09J, 041D09K, 041D09Q, 041D09R, 041D0A6, 041D0A7, 041D0A8, 041D0A9, 041D0AB, 041D0AC, 041D0AD, 041D0AF, 041D0AG, 041D0AH, 041D0AJ, 041D0AK, 041D0AQ, 041D0AR, 041D0J6, 041D0J7, 041D0J8, 041D0J9, 041D0JB, 041D0JC, 041D0JD, 041D0JF, 041D0JG, 041D0JH, 041D0JJ, 041D0JK, 041D0JQ, 041D0JR, 041D0K6, 041D0K7, 041D0K8, 041D0K9, 041D0KB, 041D0KC, 041D0KD, 041D0KF, 041D0KG, 041D0KH, 041D0KJ, 041D0KK, 041D0KQ, 041D0KR, 041D0Z6, 041D0Z7, 041D0Z8, 041D0Z9, 041D0ZB, 041D0ZC, 041D0ZD, 041D0ZF, 041D0ZG, 041D0ZH, 041D0ZJ, 041D0ZK, 041D0ZQ, 041D0ZR, 041D490, 041D496, 041D497, 041D498, 041D499, 041D49B, 041D49C, 041D49D, 041D49F, 041D49G, 041D49H, 041D49J, 041D49K, 041D49Q, 041D49R, 041D4A0, 041D4A6, 041D4A7, 041D4A8, 041D4A9, 041D4AB, 041D4AC, 041D4AD, 041D4AF, 041D4AG, 041D4AH, 041D4AJ, 041D4AK, 041D4AQ, 041D4AR, 041D4J0, 041D4J6, 041D4J7, 041D4J8, 041D4J9, 041D4JB, 041D4JC, 041D4JD, 041D4JF, 041D4JG, 041D4JH, 041D4JJ, 041D4JK, 041D4JQ, 041D4JR, 041D4K0, 041D4K6, 041D4K7, 041D4K8, 041D4K9, 041D4KB, 041D4KC, 041D4KD, 041D4KF, 041D4KG, 041D4KH, 041D4KJ, 041D4KK, 041D4KQ, 041D4KR, 041D4Z0, 041D4Z6, 041D4Z7, 041D4Z8, 041D4Z9, 041D4ZB, 041D4ZC, 041D4ZD, 041D4ZF, 041D4ZG, 041D4ZH, 041D4ZJ, 041D4ZK, 041D4ZQ, 041D4ZR, 041E099, 041E09B, 041E09C, 041E09D, 041E09F, 041E09G, 041E09H, 041E09J, 041E09K, 041E09P, 041E09Q, 041E0A9, 041E0AB, 041E0AC, 041E0AD, 041E0AF, 041E0AG, 041E0AH, 041E0AJ, 041E0AK, 041E0AP, 041E0AQ, 041E0J9, 041E0JB, 041E0JC, 041E0JD, 041E0JF, 041E0JG, 041E0JH, 041E0JJ, 041E0JK, 041E0JP, 041E0JQ, 041E0K9, 041E0KB, 041E0KC, 041E0KD, 041E0KF, 041E0KG, 041E0KH, 041E0KJ, 041E0KK, 041E0KP, 041E0KQ, 041E0Z9, 041E0ZB, 041E0ZC, 041E0ZD, 041E0ZF, 041E0ZG, 041E0ZH, 041E0ZJ, 041E0ZK, 041E0ZP, 041E0ZQ, 041E499, 041E49B, 041E49C, 041E49D, 041E49F, 041E49G, 041E49H, 041E49J, 041E49K, 041E49P, 041E49Q, 041E4A9, 041E4AB, 041E4AC, 041E4AD, 041E4AF, 041E4AG, 041E4AH, 041E4AJ, 041E4AK, 041E4AP, 041E4AQ, 041E4J9, 041E4JB, 041E4JC, 041E4JD, 041E4JF, 041E4JG, 041E4JH, 041E4JJ, 041E4JK, 041E4JP, 041E4JQ, 041E4K9, 041E4KB, 041E4KC, 041E4KD, 041E4KF, 041E4KG, 041E4KH, 041E4KJ, 041E4KK, 041E4KP, 041E4KQ, 041E4Z9, 041E4ZB, 041E4ZC, 041E4ZD, 041E4ZF, 041E4ZG, 041E4ZH, 041E4ZJ, 041E4ZK, 041E4ZP,

© 2022 Majmundar M et al. *JAMA Network Open.*

041E4ZQ, 041F099, 041F09B, 041F09C, 041F09D, 041F09F, 041F09G, 041F09H, 041F09J, 041F09K, 041F09P, 041F09Q, 041F0A9, 041F0AB, 041F0AC, 041F0AD, 041F0AF, 041F0AG, 041F0AH, 041F0AJ, 041F0AK, 041F0AP, 041F0AQ, 041F0J9, 041F0JB, 041F0JC, 041F0JD, 041F0JF, 041F0JG, 041F0JH, 041F0JJ, 041F0JK, 041F0JP, 041F0JQ, 041F0K9, 041F0KB, 041F0KC, 041F0KD, 041F0KF, 041F0KG, 041F0KH, 041F0KJ, 041F0KK, 041F0KP, 041F0KQ, 041F0Z9, 041F0ZB, 041F0ZC, 041F0ZD, 041F0ZF, 041F0ZG, 041F0ZH, 041F0ZJ, 041F0ZK, 041F0ZP, 041F0ZQ, 041F499, 041F49B, 041F49C, 041F49D, 041F49F, 041F49G, 041F49H, 041F49J, 041F49K, 041F49P, 041F49Q, 041F4A9, 041F4AB, 041F4AC, 041F4AD, 041F4AF, 041F4AG, 041F4AH, 041F4AJ, 041F4AK, 041F4AP, 041F4AQ, 041F4J9, 041F4JB, 041F4JC, 041F4JD, 041F4JF, 041F4JG, 041F4JH, 041F4JJ, 041F4JK, 041F4JP, 041F4JQ, 041F4K9, 041F4KB, 041F4KC, 041F4KD, 041F4KF, 041F4KG, 041F4KH, 041F4KJ, 041F4KK, 041F4KP, 041F4KQ, 041F4Z9, 041F4ZB, 041F4ZC, 041F4ZD, 041F4ZF, 041F4ZG, 041F4ZH, 041F4ZJ, 041F4ZK, 041F4ZP, 041F4ZQ, 041H099, 041H09B, 041H09C, 041H09D, 041H09F, 041H09G, 041H09H, 041H09J, 041H09K, 041H09P, 041H09Q, 041H0A9, 041H0AB, 041H0AC, 041H0AD, 041H0AF, 041H0AG, 041H0AH, 041H0AJ, 041H0AK, 041H0AP, 041H0AQ, 041H0J9, 041H0JB, 041H0JC, 041H0JD, 041H0JF, 041H0JG, 041H0JH, 041H0JJ, 041H0JK, 041H0JP, 041H0JQ, 041H0K9, 041H0KB, 041H0KC, 041H0KD, 041H0KF, 041H0KG, 041H0KH, 041H0KJ, 041H0KK, 041H0KP, 041H0KQ, 041H0Z9, 041H0ZB, 041H0ZC, 041H0ZD, 041H0ZF, 041H0ZG, 041H0ZH, 041H0ZJ, 041H0ZK, 041H0ZP, 041H0ZQ, 041H499, 041H49B, 041H49C, 041H49D, 041H49F, 041H49G, 041H49H, 041H49J, 041H49K, 041H49P, 041H49Q, 041H4A9, 041H4AB, 041H4AC, 041H4AD, 041H4AF, 041H4AG, 041H4AH, 041H4AJ, 041H4AK, 041H4AP, 041H4AQ, 041H4J9, 041H4JB, 041H4JC, 041H4JD, 041H4JF, 041H4JG, 041H4JH, 041H4JJ, 041H4JK, 041H4JP, 041H4JQ, 041H4K9, 041H4KB, 041H4KC, 041H4KD, 041H4KF, 041H4KG, 041H4KH, 041H4KJ, 041H4KK, 041H4KP, 041H4KQ, 041H4Z9, 041H4ZB, 041H4ZC, 041H4ZD, 041H4ZF, 041H4ZG, 041H4ZH, 041H4ZJ, 041H4ZK, 041H4ZP, 041H4ZQ, 041J099, 041J09B, 041J09C, 041J09D, 041J09F, 041J09G, 041J09H, 041J09J, 041J09K, 041J09P, 041J09Q, 041J0A9, 041J0AB, 041J0AC, 041J0AD, 041J0AF, 041J0AG, 041J0AH, 041J0AJ, 041J0AK, 041J0AP, 041J0AQ, 041J0J9, 041J0JB, 041J0JC, 041J0JD, 041J0JF, 041J0JG, 041J0JH, 041J0JJ, 041J0JK, 041J0JP, 041J0JQ, 041J0K9, 041J0KB, 041J0KC, 041J0KD, 041J0KF, 041J0KG, 041J0KH, 041J0KJ, 041J0KK, 041J0KP, 041J0KQ, 041J0Z9, 041J0ZB, 041J0ZC, 041J0ZD, 041J0ZF, 041J0ZG, 041J0ZH, 041J0ZJ, 041J0ZK, 041J0ZP, 041J0ZQ, 041J499, 041J49B, 041J49C, 041J49D, 041J49F, 041J49G, 041J49H, 041J49J, 041J49K, 041J49P, 041J49Q, 041J4A9, 041J4AB, 041J4AC, 041J4AD, 041J4AF, 041J4AG, 041J4AH, 041J4AJ, 041J4AK, 041J4AP, 041J4AQ, 041J4J9, 041J4JB, 041J4JC, 041J4JD, 041J4JF, 041J4JG, 041J4JH, 041J4JJ, 041J4JK, 041J4JP, 041J4JQ, 041J4K9, 041J4KB, 041J4KC, 041J4KD, 041J4KF, 041J4KG, 041J4KH, 041J4KJ, 041J4KK, 041J4KP, 041J4KQ, 041J4Z9, 041J4ZB, 041J4ZC, 041J4ZD, 041J4ZF, 041J4ZG, 041J4ZH, 041J4ZJ, 041J4ZK, 041J4ZP, 041J4ZQ, 0312096, 0312097, 0312098, 0312099, 031209B, 031209C, 03120A6, 03120A7, 03120A8, 03120A9, 03120AB, 03120AC, 03120J6, 03120J7, 03120J8, 03120J9, 03120JB, 03120JC, 03120K6, 03120K7, 03120K8, 03120K9, 03120KB, 03120KC, 03120Z6, 03120Z7, 03120Z8, 03120Z9, 03120ZB, 03120ZC, 0313096, 0313097, 0313098, 0313099, 031309B, 031309C, 03130A6, 03130A7, 03130A8, 03130A9, 03130AB, 03130AC, 03130J6, 03130J7, 03130J8, 03130J9, 03130JB, 03130JC, 03130K6, 03130K7, 03130K8, 03130K9, 03130KB, 03130KC, 03130Z6, 03130Z7, 03130Z8, 03130Z9, 03130ZB, 03130ZC, 0314096, 0314097, 0314098, 0314099, 031409B, 031409C, 03140A6, 03140A7, 03140A8, 03140A9, 03140AB, 03140AC, 03140J6,

© 2022 Majmundar M et al. *JAMA Network Open.*

03140J7, 03140J8, 03140J9, 03140JB, 03140JC, 03140K6, 03140K7, 03140K8, 03140K9, 03140KB, 03140KC, 03140Z6, 03140Z7, 03140Z8, 03140Z9, 03140ZB, 03140ZC, 0315096, 0315097, 0315098, 0315099, 031509B, 031509C, 03150A6, 03150A7, 03150A8, 03150A9, 03150AB, 03150AC, 03150J6, 03150J7, 03150J8, 03150J9, 03150JB, 03150JC, 03150K6, 03150K7, 03150K8, 03150K9, 03150KB, 03150KC, 03150Z6, 03150Z7, 03150Z8, 03150Z9, 03150ZB, 03150ZC, 0316096, 0316097, 0316098, 0316099, 031609B, 031609C, 03160A6, 03160A7, 03160A8, 03160A9, 03160AB, 03160AC, 03160J6, 03160J7, 03160J8, 03160J9, 03160JB, 03160JC, 03160K6, 03160K7, 03160K8, 03160K9, 03160KB, 03160KC, 03160Z6, 03160Z7, 03160Z8, 03160Z9, 03160ZB, 03160ZC, 041K09H, 041K09J, 041K09K, 041K09L, 041K09M, 041K09N, 041K09P, 041K09Q, 041K09S, 041K0AH, 041K0AJ, 041K0AK, 041K0AL, 041K0AM, 041K0AN, 041K0AP, 041K0AQ, 041K0AS, 041K0JH, 041K0JJ, 041K0JK, 041K0JL, 041K0JM, 041K0JN, 041K0JP, 041K0JQ, 041K0JS, 041K0KH, 041K0KJ, 041K0KK, 041K0KL, 041K0KM, 041K0KN, 041K0KP, 041K0KQ, 041K0KS, 041K0ZH, 041K0ZJ, 041K0ZK, 041K0ZL, 041K0ZM, 041K0ZN, 041K0ZP, 041K0ZQ, 041K0ZS, 041K3JQ, 041K3JS, 041K49H, 041K49J, 041K49K, 041K49L, 041K49M, 041K49N, 041K49P, 041K49Q, 041K49S, 041K4AH, 041K4AJ, 041K4AK, 041K4AL, 041K4AM, 041K4AN, 041K4AP, 041K4AQ, 041K4AS, 041K4JH, 041K4JJ, 041K4JK, 041K4JL, 041K4JM, 041K4JN, 041K4JP, 041K4JQ, 041K4JS, 041K4KH, 041K4KJ, 041K4KK, 041K4KL, 041K4KM, 041K4KN, 041K4KP, 041K4KQ, 041K4KS, 041K4ZH, 041K4ZJ, 041K4ZK, 041K4ZL, 041K4ZM, 041K4ZN, 041K4ZP, 041K4ZQ, 041K4ZS, 041L09H, 041L09J, 041L09K, 041L09L, 041L09M, 041L09N, 041L09P, 041L09Q, 041L0AH, 041L0AJ, 041L0AK, 041L0AL, 041L0AM, 041L0AN, 041L0AP, 041L0AQ, 041L0JH, 041L0JJ, 041L0JK, 041L0JL, 041L0JM, 041L0JN, 041L0JP, 041L0JQ, 041L0KH, 041L0KJ, 041L0KK, 041L0KL, 041L0KM, 041L0KN, 041L0KP, 041L0KQ, 041L0ZH, 041L0ZJ, 041L0ZK, 041L0ZL, 041L0ZM, 041L0ZN, 041L0ZP, 041L0ZQ, 041L3JQ, 041L3JS, 041L49H, 041L49J, 041L49K, 041L49L, 041L49M, 041L49N, 041L49P, 041L49Q, 041L4AH, 041L4AJ, 041L4AK, 041L4AL, 041L4AM, 041L4AN, 041L4AP, 041L4AQ, 041L4JH, 041L4JJ, 041L4JK, 041L4JL, 041L4JM, 041L4JN, 041L4JP, 041L4JQ, 041L4KH, 041L4KJ, 041L4KK, 041L4KL, 041L4KM, 041L4KN, 041L4KP, 041L4KQ, 041L4ZH, 041L4ZJ, 041L4ZK, 041L4ZL, 041L4ZM, 041L4ZN, 041L4ZP, 041L4ZQ, 041M09L, 041M09M, 041M09P, 041M09Q, 041M0AL, 041M0AM, 041M0AP, 041M0AQ, 041M0JL, 041M0JM, 041M0JP, 041M0JQ, 041M0KL, 041M0KM, 041M0KP, 041M0KQ, 041M0ZL, 041M0ZM, 041M0ZP, 041M0ZQ, 041M3JQ, 041M49L, 041M49M, 041M49P, 041M49Q, 041M4AL, 041M4AM, 041M4AP, 041M4AQ, 041M4JL, 041M4JM, 041M4JP, 041M4JQ, 041M4KL, 041M4KM, 041M4KP, 041M4KQ, 041M4ZL, 041M4ZM, 041M4ZP, 041M4ZQ, 041N09L, 041N09M, 041N09P, 041N09Q, 041N0AL, 041N0AM, 041N0AP, 041N0AQ, 041N0JL, 041N0JM, 041N0JP, 041N0JQ, 041N0KL, 041N0KM, 041N0KP, 041N0KQ, 041N0ZL, 041N0ZM, 041N0ZP, 041N0ZQ, 041N3JQ, 041N49L, 041N49M, 041N49P, 041N49Q, 041N4AL, 041N4AM, 041N4AP, 041N4AQ, 041N4JL, 041N4JM, 041N4JP, 041N4JQ, 041N4KL, 041N4KM, 041N4KP, 041N4KQ, 041N4ZL, 041N4ZM, 041N4ZP, 041N4ZQ, 041T09P, 041T09Q, 041T0AP, 041T0AQ, 041T0JP, 041T0JQ, 041T0KP, 041T0KQ, 041T0ZP, 041T0ZQ, 041T3JQ, 041T49P, 041T49Q, 041T4AP, 041T4AQ, 041T4JP, 041T4JQ, 041T4KP, 041T4KQ, 041T4ZP, 041T4ZQ, 041U09P, 041U09Q, 041U0AP, 041U0AQ, 041U0JP, 041U0JQ, 041U0KP, 041U0KQ, 041U0ZP, 041U0ZQ, 041U3JQ, 041U49P, 041U49Q, 041U4AP, 041U4AQ, 041U4JP, 041U4JQ, 041U4KP, 041U4KQ, 041U4ZP, 041U4ZQ, 041V09P, 041V09Q, 041V0AP, 041V0AQ, 041V0JP, 041V0JQ, 041V0KP, 041V0KQ, 041V0ZP, 041V0ZQ, 041V3JQ, 041V49P, 041V49Q, 041V4AP, 041V4AQ, 041V4JP, 041V4JQ, 041V4KP,

© 2022 Majmundar M et al. *JAMA Network Open.*

041V4KQ, 041V4ZP, 041V4ZQ, 041W09P, 041W09Q, 041W0AP, 041W0AQ, 041W0JP, 041W0JQ, 041W0KP, 041W0KQ, 041W0ZP, 041W0ZQ, 041W3JQ, 041W49P, 041W49Q, 041W4AP, 041W4AQ, 041W4JP, 041W4JQ, 041W4KP, 041W4KQ, 041W4ZP, 041W4ZQ

© 2022 Majmundar M et al. *JAMA Network Open.*

**Surgical Revascularization**
**Endovascular Revascularization**

© 2022 Majmundar M et al. *JAMA Network Open.*

047C041, 047C046, 047C04Z, 047C056, 047C05Z, 047C066, 047C06Z, 047C076, 047C07Z, 047C0D1, 047C0D6, 047C0DZ, 047C0E6, 047C0EZ, 047C0F6, 047C0FZ, 047C0G6, 047C0GZ, 047C0Z1, 047C0Z6, 047C0ZZ, 047C341, 047C346, 047C34Z, 047C356, 047C35Z, 047C366, 047C36Z, 047C376, 047C37Z, 047C3D1, 047C3D6, 047C3DZ, 047C3E6, 047C3EZ, 047C3F6, 047C3FZ, 047C3G6, 047C3GZ, 047C3Z1, 047C3Z6, 047C3ZZ, 047C441, 047C446, 047C44Z, 047C456, 047C45Z, 047C466, 047C46Z, 047C476, 047C47Z, 047C4D1, 047C4D6, 047C4DZ, 047C4E6, 047C4EZ, 047C4F6, 047C4FZ, 047C4G6, 047C4GZ, 047C4Z1, 047C4Z6, 047C4ZZ, 047D041, 047D046, 047D04Z, 047D056, 047D05Z, 047D066, 047D06Z, 047D076, 047D07Z, 047D0D1, 047D0D6, 047D0DZ, 047D0E6, 047D0EZ, 047D0F6, 047D0FZ, 047D0G6, 047D0GZ, 047D0Z1, 047D0Z6, 047D0ZZ, 047D341, 047D346, 047D34Z, 047D356, 047D35Z, 047D366, 047D36Z, 047D376, 047D37Z, 047D3D1, 047D3D6, 047D3DZ, 047D3E6, 047D3EZ, 047D3F6, 047D3FZ, 047D3G6, 047D3GZ, 047D3Z1, 047D3Z6, 047D3ZZ, 047D441, 047D446, 047D44Z, 047D456, 047D45Z, 047D466, 047D46Z, 047D476, 047D47Z, 047D4D1, 047D4D6, 047D4DZ, 047D4E6, 047D4EZ, 047D4F6, 047D4FZ, 047D4G6, 047D4GZ, 047D4Z1, 047D4Z6, 047D4ZZ, 047E041, 047E046, 047E04Z, 047E056, 047E05Z, 047E066, 047E06Z, 047E076, 047E07Z, 047E0D1, 047E0D6, 047E0DZ, 047E0E6, 047E0EZ, 047E0F6, 047E0FZ, 047E0G6, 047E0GZ, 047E0Z1, 047E0Z6, 047E0ZZ, 047E341, 047E346, 047E34Z, 047E356, 047E35Z, 047E366, 047E36Z, 047E376, 047E37Z, 047E3D1, 047E3D6, 047E3DZ, 047E3E6, 047E3EZ, 047E3F6, 047E3FZ, 047E3G6, 047E3GZ, 047E3Z1, 047E3Z6, 047E3ZZ, 047E441, 047E446, 047E44Z, 047E456, 047E45Z, 047E466, 047E46Z, 047E476, 047E47Z, 047E4D1, 047E4D6, 047E4DZ, 047E4E6, 047E4EZ, 047E4F6, 047E4FZ, 047E4G6, 047E4GZ, 047E4Z1, 047E4Z6, 047E4ZZ, 047F041, 047F046, 047F04Z, 047F056, 047F05Z, 047F066, 047F06Z, 047F076, 047F07Z, 047F0D1, 047F0D6, 047F0DZ, 047F0E6, 047F0EZ, 047F0F6, 047F0FZ, 047F0G6, 047F0GZ, 047F0Z1, 047F0Z6, 047F0ZZ, 047F341, 047F346, 047F34Z, 047F356, 047F35Z, 047F366, 047F36Z, 047F376, 047F37Z, 047F3D1, 047F3D6, 047F3DZ, 047F3E6, 047F3EZ, 047F3F6, 047F3FZ, 047F3G6, 047F3GZ, 047F3Z1, 047F3Z6, 047F3ZZ, 047F441, 047F446, 047F44Z, 047F456, 047F45Z, 047F466, 047F46Z, 047F476, 047F47Z, 047F4D1, 047F4D6, 047F4DZ, 047F4E6, 047F4EZ, 047F4F6, 047F4FZ, 047F4G6, 047F4GZ, 047F4Z1, 047F4Z6, 047F4ZZ, 047H041, 047H046, 047H04Z, 047H056, 047H05Z, 047H066, 047H06Z, 047H076, 047H07Z, 047H0D1, 047H0D6, 047H0DZ, 047H0E6, 047H0EZ, 047H0F6, 047H0FZ, 047H0G6, 047H0GZ, 047H0Z1, 047H0Z6, 047H0ZZ, 047H341, 047H346, 047H34Z, 047H356, 047H35Z, 047H366, 047H36Z, 047H376, 047H37Z, 047H3D1, 047H3D6, 047H3DZ, 047H3E6, 047H3EZ, 047H3F6, 047H3FZ, 047H3G6, 047H3GZ, 047H3Z1, 047H3Z6, 047H3ZZ, 047H441, 047H446, 047H44Z, 047H456, 047H45Z, 047H466, 047H46Z, 047H476, 047H47Z, 047H4D1, 047H4D6, 047H4DZ, 047H4E6, 047H4EZ, 047H4F6, 047H4FZ, 047H4G6, 047H4GZ, 047H4Z1, 047H4Z6, 047H4ZZ, 047J041, 047J046, 047J04Z, 047J056, 047J05Z, 047J066, 047J06Z, 047J076, 047J07Z, 047J0D1, 047J0D6, 047J0DZ, 047J0E6, 047J0EZ, 047J0F6, 047J0FZ, 047J0G6, 047J0GZ, 047J0Z1, 047J0Z6, 047J0ZZ, 047J341, 047J346, 047J34Z, 047J356, 047J35Z, 047J366, 047J36Z, 047J376, 047J37Z, 047J3D1, 047J3D6, 047J3DZ, 047J3E6, 047J3EZ, 047J3F6, 047J3FZ, 047J3G6, 047J3GZ, 047J3Z1, 047J3Z6, 047J3ZZ, 047J441, 047J446, 047J44Z, 047J456, 047J45Z, 047J466, 047J46Z, 047J476, 047J47Z, 047J4D1, 047J4D6, 047J4DZ, 047J4E6, 047J4EZ, 047J4F6, 047J4FZ, 047J4G6, 047J4GZ, 047J4Z1, 047J4Z6, 047J4ZZ, 047K041, 047K046, 047K04Z, 047K056, 047K05Z, 047K066, 047K06Z, 047K076, 047K07Z, 047K0D1, 047K0D6, 047K0DZ, 047K0E6, 047K0EZ, 047K0F6, 047K0FZ, 047K0G6, 047K0GZ, 047K0Z1, 047K0Z6, 047K0ZZ,

© 2022 Majmundar M et al. *JAMA Network Open.*

047K341, 047K346, 047K34Z, 047K356, 047K35Z, 047K366, 047K36Z, 047K376, 047K37Z, 047K3D1, 047K3D6, 047K3DZ, 047K3E6, 047K3EZ, 047K3F6, 047K3FZ, 047K3G6, 047K3GZ, 047K3Z1, 047K3Z6, 047K3ZZ, 047K441, 047K446, 047K44Z, 047K456, 047K45Z, 047K466, 047K46Z, 047K476, 047K47Z, 047K4D1, 047K4D6, 047K4DZ, 047K4E6, 047K4EZ, 047K4F6, 047K4FZ, 047K4G6, 047K4GZ, 047K4Z1, 047K4Z6, 047K4ZZ, 047L041, 047L046, 047L04Z, 047L056, 047L05Z, 047L066, 047L06Z, 047L076, 047L07Z, 047L0D1, 047L0D6, 047L0DZ, 047L0E6, 047L0EZ, 047L0F6, 047L0FZ, 047L0G6, 047L0GZ, 047L0Z1, 047L0Z6, 047L0ZZ, 047L341, 047L346, 047L34Z, 047L356, 047L35Z, 047L366, 047L36Z, 047L376, 047L37Z, 047L3D1, 047L3D6, 047L3DZ, 047L3E6, 047L3EZ, 047L3F6, 047L3FZ, 047L3G6, 047L3GZ, 047L3Z1, 047L3Z6, 047L3ZZ, 047L441, 047L446, 047L44Z, 047L456, 047L45Z, 047L466, 047L46Z, 047L476, 047L47Z, 047L4D1, 047L4D6, 047L4DZ, 047L4E6, 047L4EZ, 047L4F6, 047L4FZ, 047L4G6, 047L4GZ, 047L4Z1, 047L4Z6, 047L4ZZ, 047M041, 047M046, 047M04Z, 047M056, 047M05Z, 047M066, 047M06Z, 047M076, 047M07Z, 047M0D1, 047M0D6, 047M0DZ, 047M0E6, 047M0EZ, 047M0F6, 047M0FZ, 047M0G6, 047M0GZ, 047M0Z1, 047M0Z6, 047M0ZZ, 047M341, 047M346, 047M34Z, 047M356, 047M35Z, 047M366, 047M36Z, 047M376, 047M37Z, 047M3D1, 047M3D6, 047M3DZ, 047M3E6, 047M3EZ, 047M3F6, 047M3FZ, 047M3G6, 047M3GZ, 047M3Z1, 047M3Z6, 047M3ZZ, 047M441, 047M446, 047M44Z, 047M456, 047M45Z, 047M466, 047M46Z, 047M476, 047M47Z, 047M4D1, 047M4D6, 047M4DZ, 047M4E6, 047M4EZ, 047M4F6, 047M4FZ, 047M4G6, 047M4GZ, 047M4Z1, 047M4Z6, 047M4ZZ, 047N041, 047N046, 047N04Z, 047N056, 047N05Z, 047N066, 047N06Z, 047N076, 047N07Z, 047N0D1, 047N0D6, 047N0DZ, 047N0E6, 047N0EZ, 047N0F6, 047N0FZ, 047N0G6, 047N0GZ, 047N0Z1, 047N0Z6, 047N0ZZ, 047N341, 047N346, 047N34Z, 047N356, 047N35Z, 047N366, 047N36Z, 047N376, 047N37Z, 047N3D1, 047N3D6, 047N3DZ, 047N3E6, 047N3EZ, 047N3F6, 047N3FZ, 047N3G6, 047N3GZ, 047N3Z1, 047N3Z6, 047N3ZZ, 047N441, 047N446, 047N44Z, 047N456, 047N45Z, 047N466, 047N46Z, 047N476, 047N47Z, 047N4D1, 047N4D6, 047N4DZ, 047N4E6, 047N4EZ, 047N4F6, 047N4FZ, 047N4G6, 047N4GZ, 047N4Z1, 047N4Z6, 047N4ZZ, 047P041, 047P046, 047P04Z, 047P056, 047P05Z, 047P066, 047P06Z, 047P076, 047P07Z, 047P0D1, 047P0D6, 047P0DZ, 047P0E6, 047P0EZ, 047P0F6, 047P0FZ, 047P0G6, 047P0GZ, 047P0Z1, 047P0Z6, 047P0ZZ, 047P341, 047P346, 047P34Z, 047P356, 047P35Z, 047P366, 047P36Z, 047P376, 047P37Z, 047P3D1, 047P3D6, 047P3DZ, 047P3E6, 047P3EZ, 047P3F6, 047P3FZ, 047P3G6, 047P3GZ, 047P3Z1, 047P3Z6, 047P3ZZ, 047P441, 047P446, 047P44Z, 047P456, 047P45Z, 047P466, 047P46Z, 047P476, 047P47Z, 047P4D1, 047P4D6, 047P4DZ, 047P4E6, 047P4EZ, 047P4F6, 047P4FZ, 047P4G6, 047P4GZ, 047P4Z1, 047P4Z6, 047P4ZZ, 047Q041, 047Q046, 047Q04Z, 047Q056, 047Q05Z, 047Q066, 047Q06Z, 047Q076, 047Q07Z, 047Q0D1, 047Q0D6, 047Q0DZ, 047Q0E6, 047Q0EZ, 047Q0F6, 047Q0FZ, 047Q0G6, 047Q0GZ, 047Q0Z1, 047Q0Z6, 047Q0ZZ, 047Q341, 047Q346, 047Q34Z, 047Q356, 047Q35Z, 047Q366, 047Q36Z, 047Q376, 047Q37Z, 047Q3D1, 047Q3D6, 047Q3DZ, 047Q3E6, 047Q3EZ, 047Q3F6, 047Q3FZ, 047Q3G6, 047Q3GZ, 047Q3Z1, 047Q3Z6, 047Q3ZZ, 047Q441, 047Q446, 047Q44Z, 047Q456, 047Q45Z, 047Q466, 047Q46Z, 047Q476, 047Q47Z, 047Q4D1, 047Q4D6, 047Q4DZ, 047Q4E6, 047Q4EZ, 047Q4F6, 047Q4FZ, 047Q4G6, 047Q4GZ, 047Q4Z1, 047Q4Z6, 047Q4ZZ, 047R041, 047R046, 047R04Z, 047R056, 047R05Z, 047R066, 047R06Z, 047R076, 047R07Z, 047R0D1, 047R0D6, 047R0DZ, 047R0E6, 047R0EZ, 047R0F6, 047R0FZ, 047R0G6, 047R0GZ, 047R0Z1, 047R0Z6, 047R0ZZ, 047R341, 047R346, 047R34Z, 047R356, 047R35Z, 047R366, 047R36Z, 047R376, 047R37Z, 047R3D1, 047R3D6, 047R3DZ, 047R3E6, 047R3EZ, 047R3F6,

© 2022 Majmundar M et al. *JAMA Network Open.*

047R3FZ, 047R3G6, 047R3GZ, 047R3Z1, 047R3Z6, 047R3ZZ, 047R441, 047R446, 047R44Z, 047R456, 047R45Z, 047R466, 047R46Z, 047R476, 047R47Z, 047R4D1, 047R4D6, 047R4DZ, 047R4E6, 047R4EZ, 047R4F6, 047R4FZ, 047R4G6, 047R4GZ, 047R4Z1, 047R4Z6, 047R4ZZ, 047S041, 047S046, 047S04Z, 047S056, 047S05Z, 047S066, 047S06Z, 047S076, 047S07Z, 047S0D1, 047S0D6, 047S0DZ, 047S0E6, 047S0EZ, 047S0F6, 047S0FZ, 047S0G6, 047S0GZ, 047S0Z1, 047S0Z6, 047S0ZZ, 047S341, 047S346, 047S34Z, 047S356, 047S35Z, 047S366, 047S36Z, 047S376, 047S37Z, 047S3D1, 047S3D6, 047S3DZ, 047S3E6, 047S3EZ, 047S3F6, 047S3FZ, 047S3G6, 047S3GZ, 047S3Z1, 047S3Z6, 047S3ZZ, 047S441, 047S446, 047S44Z, 047S456, 047S45Z, 047S466, 047S46Z, 047S476, 047S47Z, 047S4D1, 047S4D6, 047S4DZ, 047S4E6, 047S4EZ, 047S4F6, 047S4FZ, 047S4G6, 047S4GZ, 047S4Z1, 047S4Z6, 047S4ZZ, 047T041, 047T046, 047T04Z, 047T056, 047T05Z, 047T066, 047T06Z, 047T076, 047T07Z, 047T0D1, 047T0D6, 047T0DZ, 047T0E6, 047T0EZ, 047T0F6, 047T0FZ, 047T0G6, 047T0GZ, 047T0Z1, 047T0Z6, 047T0ZZ, 047T341, 047T346, 047T34Z, 047T356, 047T35Z, 047T366, 047T36Z, 047T376, 047T37Z, 047T3D1, 047T3D6, 047T3DZ, 047T3E6, 047T3EZ, 047T3F6, 047T3FZ, 047T3G6, 047T3GZ, 047T3Z1, 047T3Z6, 047T3ZZ, 047T441, 047T446, 047T44Z, 047T456, 047T45Z, 047T466, 047T46Z, 047T476, 047T47Z, 047T4D1, 047T4D6, 047T4DZ, 047T4E6, 047T4EZ, 047T4F6, 047T4FZ, 047T4G6, 047T4GZ, 047T4Z1, 047T4Z6, 047T4ZZ, 047U041, 047U046, 047U04Z, 047U056, 047U05Z, 047U066, 047U06Z, 047U076, 047U07Z, 047U0D1, 047U0D6, 047U0DZ, 047U0E6, 047U0EZ, 047U0F6, 047U0FZ, 047U0G6, 047U0GZ, 047U0Z1, 047U0Z6, 047U0ZZ, 047U341, 047U346, 047U34Z, 047U356, 047U35Z, 047U366, 047U36Z, 047U376, 047U37Z, 047U3D1, 047U3D6, 047U3DZ, 047U3E6, 047U3EZ, 047U3F6, 047U3FZ, 047U3G6, 047U3GZ, 047U3Z1, 047U3Z6, 047U3ZZ, 047U441, 047U446, 047U44Z, 047U456, 047U45Z, 047U466, 047U46Z, 047U476, 047U47Z, 047U4D1, 047U4D6, 047U4DZ, 047U4E6, 047U4EZ, 047U4F6, 047U4FZ, 047U4G6, 047U4GZ, 047U4Z1, 047U4Z6, 047U4ZZ, 047V041, 047V046, 047V04Z, 047V056, 047V05Z, 047V066, 047V06Z, 047V076, 047V07Z, 047V0D1, 047V0D6, 047V0DZ, 047V0E6, 047V0EZ, 047V0F6, 047V0FZ, 047V0G6, 047V0GZ, 047V0Z1, 047V0Z6, 047V0ZZ, 047V341, 047V346, 047V34Z, 047V356, 047V35Z, 047V366, 047V36Z, 047V376, 047V37Z, 047V3D1, 047V3D6, 047V3DZ, 047V3E6, 047V3EZ, 047V3F6, 047V3FZ, 047V3G6, 047V3GZ, 047V3Z1, 047V3Z6, 047V3ZZ, 047V441, 047V446, 047V44Z, 047V456, 047V45Z, 047V466, 047V46Z, 047V476, 047V47Z, 047V4D1, 047V4D6, 047V4DZ, 047V4E6, 047V4EZ, 047V4F6, 047V4FZ, 047V4G6, 047V4GZ, 047V4Z1, 047V4Z6, 047V4ZZ, 047W041, 047W046, 047W04Z, 047W056, 047W05Z, 047W066, 047W06Z, 047W076, 047W07Z, 047W0D1, 047W0D6, 047W0DZ, 047W0E6, 047W0EZ, 047W0F6, 047W0FZ, 047W0G6, 047W0GZ, 047W0Z1, 047W0Z6, 047W0ZZ, 047W341, 047W346, 047W34Z, 047W356, 047W35Z, 047W366, 047W36Z, 047W376, 047W37Z, 047W3D1, 047W3D6, 047W3DZ, 047W3E6, 047W3EZ, 047W3F6, 047W3FZ, 047W3G6, 047W3GZ, 047W3Z1, 047W3Z6, 047W3ZZ, 047W441, 047W446, 047W44Z, 047W456, 047W45Z, 047W466, 047W46Z, 047W476, 047W47Z, 047W4D1, 047W4D6, 047W4DZ, 047W4E6, 047W4EZ, 047W4F6, 047W4FZ, 047W4G6, 047W4GZ, 047W4Z1, 047W4Z6, 047W4ZZ, 047Y041, 047Y046, 047Y04Z, 047Y056, 047Y05Z, 047Y066, 047Y06Z, 047Y076, 047Y07Z, 047Y0D1, 047Y0D6, 047Y0DZ, 047Y0E6, 047Y0EZ, 047Y0F6, 047Y0FZ, 047Y0G6, 047Y0GZ, 047Y0Z1, 047Y0Z6, 047Y0ZZ, 047Y341, 047Y346, 047Y34Z, 047Y356, 047Y35Z, 047Y366, 047Y36Z, 047Y376, 047Y37Z, 047Y3D1, 047Y3D6, 047Y3DZ, 047Y3E6, 047Y3EZ, 047Y3F6, 047Y3FZ, 047Y3G6, 047Y3GZ, 047Y3Z1, 047Y3Z6, 047Y3ZZ, 047Y441, 047Y446, 047Y44Z, 047Y456, 047Y45Z, 047Y466, 047Y46Z, 047Y476,

© 2022 Majmundar M et al. *JAMA Network Open.*

047Y47Z, 047Y4D1, 047Y4D6, 047Y4DZ, 047Y4E6, 047Y4EZ, 047Y4F6, 047Y4FZ, 047Y4G6, 047Y4GZ, 047Y4Z1, 047Y4Z6, 047Y4ZZ, 04CC0Z6, 04CC0ZZ, 04CC3Z6, 04CC3ZZ, 04CC4Z6, 04CC4ZZ, 04CD0Z6, 04CD0ZZ, 04CD3Z6, 04CD3ZZ, 04CD4Z6, 04CD4ZZ, 04CE0Z6, 04CE0ZZ, 04CE3Z6, 04CE3ZZ, 04CE4Z6, 04CE4ZZ, 04CF0Z6, 04CF0ZZ, 04CF3Z6, 04CF3ZZ, 04CF4Z6, 04CF4ZZ, 04CH0Z6, 04CH0ZZ, 04CH3Z6, 04CH3ZZ, 04CH4Z6, 04CH4ZZ, 04CJ0Z6, 04CJ0ZZ, 04CJ3Z6, 04CJ3ZZ, 04CJ4Z6, 04CJ4ZZ, 04CK0Z6, 04CK0ZZ, 04CK3Z6, 04CK3ZZ, 04CK4Z6, 04CK4ZZ, 04CL0Z6, 04CL0ZZ, 04CL3Z6, 04CL3ZZ, 04CL4Z6, 04CL4ZZ, 04CM0Z6, 04CM0ZZ, 04CM3Z6, 04CM3ZZ, 04CM4Z6, 04CM4ZZ, 04CN0Z6, 04CN0ZZ, 04CN3Z6, 04CN3ZZ, 04CN4Z6, 04CN4ZZ, 04CP0Z6, 04CP0ZZ, 04CP3Z6, 04CP3ZZ, 04CP4Z6, 04CP4ZZ, 04CQ0Z6, 04CQ0ZZ, 04CQ3Z6, 04CQ3ZZ, 04CQ4Z6, 04CQ4ZZ, 04CR0Z6, 04CR0ZZ, 04CR3Z6, 04CR3ZZ, 04CR4Z6, 04CR4ZZ, 04CS0Z6, 04CS0ZZ, 04CS3Z6, 04CS3ZZ, 04CS4Z6, 04CS4ZZ, 04CT0Z6, 04CT0ZZ, 04CT3Z6, 04CT3ZZ, 04CT4Z6, 04CT4ZZ, 04LE4CT, 04CU0Z6, 04CU0ZZ, 04CU3Z6, 04CU3ZZ, 04CU4Z6, 04CU4ZZ, 04CV0Z6, 04CV0ZZ, 04CV3Z6, 04CV3ZZ, 04CV4Z6, 04CV4ZZ, 04CW0Z6, 04CW0ZZ, 04CW3Z6, 04CW3ZZ, 04CW4Z6, 04CW4ZZ, 04CY0Z6, 04CY0ZZ, 04CY3Z6, 04CY3ZZ, 04CY4Z6, 04CY4ZZ

© 2022 Majmundar M et al. *JAMA Network Open.*

eTable 2. *ICD-10* Procedure Codes of Devices and Procedures Used for Endovascular Revascularization

Devices and Procedures	ICD 10 PCS codes
Drug-coated balloon (DCB)	047K3Z1, 047L3Z1, 047M3Z1, 047N3Z1, 047P3Z1, 047Q3Z1, 047R3Z1, 047S3Z1, 047T3Z1, 047U3Z1, 047V3Z1, 047W3Z1
Drug-coated balloon (DCB) + drug-eluting stent (DES)	047K341, 047L341, 047M341, 047N341, 047P341, 047Q341, 047R341, 047S341, 047T341, 047U341, 047V341, 047W341
Drug-coated balloon (DCB) + bare metal stent (BMS)	047K3D1, 047L3D1, 047M3D1, 047N3D1, 047P3D1, 047Q3D1, 047R3D1, 047S3D1, 047T3D1, 047U3D1, 047V3D1, 047W3D1
Uncoated Percutaneous Transluminal Angioplasty Balloon (PTA)	047K3Z6, 047K3ZZ, 047L3ZZ, 047L3Z6, 047M3Z6, 047M3ZZ, 047N3Z6, 047N3ZZ, 047P3Z6, 047P3ZZ, 047Q3Z6, 047Q3ZZ, 047R3Z6, 047R3ZZ, 047S3Z6, 047S3ZZ, 047T3Z6, 047T3ZZ, 047U3Z6, 047U3ZZ, 047V3Z6, 047V3ZZ, 047W3Z6, 047W3ZZ
Drug-eluting stent (DES)	047K346, 047K34Z, 047K356, 047K35Z, 047K366, 047K36Z, 047K376, 047K37Z, 047L346, 047L34Z, 047L356, 047L35Z, 047L366, 047L36Z, 047L376, 047L37Z, 047M346, 047M34Z, 047M356, 047M35Z, 047M366, 047M36Z, 047M376, 047M37Z, 047N346, 047N34Z, 047N356, 047N35Z, 047N366, 047N36Z, 047N376, 047N37Z, 047P346, 047P356, 047P366, 047P376, 047Q346, 047Q356, 047Q366, 047Q376, 047R346, 047R356, 047R366, 047R376, 047S346, 047S356, 047S366, 047S376, 047T346, 047T356, 047T366, 047T376, 047U346, 047U356, 047U366, 047U376, 047V346, 047V356, 047V366, 047V376, 047W346, 047W356, 047W366, 047W376, 047P34Z, 047P35Z, 047P36Z, 047P37Z, 047Q34Z, 047Q35Z, 047Q36Z, 047Q37Z, 047R34Z, 047R35Z, 047R36Z, 047R37Z, 047S34Z, 047S35Z, 047S36Z, 047S37Z, 047T34Z, 047T35Z, 047T36Z, 047T37Z, 047U34Z, 047U35Z, 047U36Z, 047U37Z, 047V34Z, 047V35Z, 047V36Z, 047V37Z, 047W34Z, 047W35Z, 047W36Z, 047W37Z

© 2022 Majmundar M et al. *JAMA Network Open.*

Devices and Procedures	ICD 10 PCS codes
Bare metal stent (BMS)	047K3D6, 047K3DZ, 047K3E6, 047K3EZ, 047K3F6, 047K3FZ, 047K3G6, 047K3GZ, 047L3D6, 047L3DZ, 047L3E6, 047L3EZ, 047L3F6, 047L3FZ, 047L3G6, 047L3GZ, 047M3D6, 047M3DZ, 047M3E6, 047M3EZ, 047M3F6, 047M3FZ, 047M3G6, 047M3GZ, 047N3D6, 047N3DZ, 047N3E6, 047N3EZ, 047N3F6, 047N3FZ, 047N3G6, 047N3GZ, 047P3D6, 047P3E6, 047P3F6, 047P3G6, 047Q3D6, 047Q3E6, 047Q3F6, 047Q3G6, 047R3D6, 047R3E6, 047R3F6, 047R3G6, 047S3D6, 047S3E6, 047S3F6, 047S3G6, 047T3D6, 047T3E6, 047T3F6, 047T3G6, 047U3D6, 047U3E6, 047U3F6, 047U3G6, 047V3D6, 047V3E6, 047V3F6, 047V3G6, 047W3D6, 047W3E6, 047W3F6, 047W3G6, 047P3DZ, 047P3EZ, 047P3FZ, 047P3GZ, 047Q3DZ, 047Q3EZ, 047Q3FZ, 047Q3GZ, 047R3DZ, 047R3EZ, 047R3FZ, 047R3GZ, 047S3DZ, 047S3EZ, 047S3FZ, 047S3GZ, 047T3DZ, 047T3EZ, 047T3FZ, 047T3GZ, 047U3DZ, 047U3EZ, 047U3FZ, 047U3GZ, 047V3DZ, 047V3EZ, 047V3FZ, 047V3GZ, 047W3DZ, 047W3EZ, 047W3FZ, 047W3GZ
Atherectomy	04CK3ZZ, 04CL3ZZ, 04CM3ZZ, 04CN3ZZ, 04CP3ZZ, 04CQ3ZZ, 04CR3ZZ, 04CS3ZZ, 04CT3ZZ, 04CU3ZZ, 04CV3ZZ, 04CW3ZZ

© 2022 Majmundar M et al. *JAMA Network Open.*

eTable 3. *ICD-10* Codes of Variables Used for Clinical Presentation and Comorbidities

**Clinical Presentation**	
Rest pain	I70.22
Ulceration	I70.23, I70.24, I70.25
Gangrene	I70.26, I96
Osteomyelitis lower extremity	M86.00, M86.05, M86.06, M86.07, M86.08, M86.09, M86.10, M86.15, M86.16, M86.17, M86.18, M86.19, M86.20, M86.25, M86.26, M86.27, M86.28, M86.29, M86.30, M86.35, M86.36, M86.37, M86.38, M86.39, M86.40, M86.45, M86.46, M86.47, M86.48, M86.49, M86.50, M86.55, M86.56, M86.57, M86.58, M86.59, M86.60, M86.65, M86.66, M86.67, M86.68, M86.69, M86.8X0, M86.8X5, M86.8X6, M86.8X7, M86.8X8, M86.8X9
Atherosclerosis of aorta	I70.0
Chronic total occlusion	I70.92
Generalized atherosclerosis	I70.91
Nontraumatic ischemic infarction of lower muscle	M62.25, M62.26, M62.27
Sepsis	A40, A41, R65.20, R65.21
Bacteremia	R78.81
History of prior lower extremity amputation	Z894, Z895, Z896, Z899
Impaired mobility	Z74, Z993, R26
Oxygen dependent	Z998
**Comorbidities**	
Smoking	F17200, F17201, F17203, F17208, F17209, F17210, F17211,F17213, F17218, F17219, F17220, F17290, F17299, Z87891
Carotid artery disease	I652
End-stage renal disease	N186
History of non-adherence	Z9112, Z9113, Z9114, Z9119
Prior PCI	Z98.61
Prior CABG	Z95.1

© 2022 Majmundar M et al. *JAMA Network Open.*

Ischemic heart disease	I24.8, I24.9, I25.1, I25.10, I25.11, I25.110, I25.111, I25.118, I25.119, I25.2, I25.5, I25.6, I25.8, I25.810, I25.89, I25.9, I25.82, I25.83, I25.84, I25.41, I25.42, I25.700, I25.701, I25.708, I25.709, I25.710, I25.711, I25.718, I25.719, I25.720, I25.721, I25.728, I25.729, I25.730, I25.731, I25.738, I25.739, I25.790, I25.791, I25.798, I25.799
Stroke/TIA	I69.3, Z86.73
Atrial fibrillation	I48, I48.0, I48.1, I48.2, I48.4, I48.91

© 2022 Majmundar M et al. *JAMA Network Open.*

eTable 4. *ICD-10* Codes for Outcomes

**Major amputation**	0Y6M0Z0, 0Y6N0Z0, 0Y6C0Z1, 0Y6C0Z2, 0Y6C0Z3, 0Y6D0Z1, 0Y6D0Z2, 0Y6D0Z3, 0Y6H0Z1, 0Y6H0Z2, 0Y6H0Z3, 0Y6J0Z1, 0Y6J0Z2, 0Y6J0Z3, 0Y6F0ZZ, 0Y6G0ZZ, 0Y670ZZ, 0Y680ZZ, 0Y620ZZ, 0Y630ZZ, 0Y640ZZ
**AKI**	N170, N171, N172, N178, N179, N19, N990, R34
**Major bleeding**	**Bleeding "AND" blood transfusion**
**Blood transfusion**	30243N0 30243N1 30243P0 30243P1 30243H0 30243H1 30240N0 30240N1 30240P0 30240P1 30240H0 30240H1 30230H0 30230H1 30230N0 30230N1 30230P0 30230P1 30233N0 30233N1 30233P0 30233P1
**Bleeding**	
Postoperative hemorrhage or hematoma	I97418, I97618, I97620, I97621, I97638, D62, L7602, L7622, L7632, M96811, M96831, M96841
Hemoperitoneum	K66.1
GI bleed	K2211, K250, K252, K254, K256, K2901, K2921, K2931, K2941, K2951, K2961, K2971, K2981, K2991, K260, K262, K264, K266, K270, K272, K274, K276, K5701, K5711, K5713, K5721, K5731, K5733, K5741, K5751, K5753, K5781, K5791, K5793, K51011, K51211, K51311, K51411, K51511, K51811, K51911, K50011, K50111, K50811, K50911, K625, K5521
GU bleed	R31.0, R31.9
Hemoptysis	R04.2
Epistaxis	R04.0
Unspecified hemorrhage	R58
Intracranial bleed	I60, I61, I62, I690, I691, I692
**Vascular complication**	T817,<br>S15, S25, S35, S45, S55, S65, S75, S85, S95, S090<br>02Q, 03Q, 04Q, 05Q, 06Q, 0GQ6, 0GQ7, 0GQ8, 0GQ9, 0GQD, 03L, 04L<br>I77.0

© 2022 Majmundar M et al. *JAMA Network Open.*

**Myocardial infarction**	I21, I22, I200, I25110, I240, I248
**Acute stroke**	
Intracranial bleed	I60, I61, I62, I690, I691, I692
Systemic embolism	I63, G46
Hemorrhagic stroke	I61, I629
Postoperative stroke or TIA	I97810, I97811, I97820, I97821

© 2022 Majmundar M et al. *JAMA Network Open.*

eTable 5. Details About Time-to-Event Analysis

Month of admission	Follow-up duration of an event other than mortality[#]	Follow-up duration of mortality during readmission[µ]	Follow-up without an event*
January	(Time of readmission) - (Time of index admission + length of stay on index admission)	(Time of readmission + length of stay of readmission) – (Time of index admission + length of stay of index admission)	335-365
February			307-334
March			276-306
April			246-275
May			215-245
June			185-214
July			154-184
August			123-153
September			93-122
October			62-92
November			32-61
December			0-31

Time between discharge day of index admission to the first day of readmission.
µ Time between discharge day of index admission to the day patient died during readmission.
*Randomly assigned.
We utilized NRD_visitlink and NRD_daystoevent to track patients across the hospital over follow-up duration. Details are in following links (https://www.hcup-us.ahrq.gov/db/vars/nrd_visitlink/nrdnote.jsp and https://www.hcup-us.ahrq.gov/db/vars/nrd_daystoevent/nrdnote.jsp)
Mortality was considered censoring event for other readmission outcomes.
MACE outcome: In case if patient had multiple events, timing of first event was considered time to event.

© 2022 Majmundar M et al. *JAMA Network Open.*

eTable 6. Studies Comparing Endovascular vs Surgical Revascularization in Patients With Critical Limb Ischemia

Trial or Study Name	Dates of Trial or Study	Study Design/Method	No. of Patients	Primary Study endpoint	Results	Other events and comments
**Adam et al,[7] 2005**	August 1999 - June 2004	Multicenter randomized trial	452 patients,rando mized 1:1 to ER or SR; 224 in ER and 228 in SR.	AFS*	Similar AFS (HR of Sr =0.73, 95% CI, 0.49 to 1.07) at 1 year.<br><br>Similar all-cause mortality (HR of SR =0.81, 95% CI, 0.55 to 1.19) between ER and SR at 1 year.	Use of balloon angioplasty in ER technique, few included patients were of acute limb ischemia
**Bisdas et al,[18] 2016**	January 2013 - September 2014	Prospective cohort study with multivariable Cox regression model	1200 patients, 642 (53%) in ER and 284 (24%) in SR	AFS at 1 year	Similar AFS (HR of ER vs SR = 0.91, 95% CI, 0.70 to 1.19, P = 0.492) at 1 year.<br><br>Similar Freedom from amputation (HR =0.86, 95% CI, 0.56 to 1.30) at 1 year.<br><br>Similar survival (HR = 1.14, 95% CI, 0.80 to 1.63) between ER and SR at 1 year.	New-onset CLI patients were included
**Lin et al,[19] 2019**	January 2005 - December 2013	Retrospective analysis with IPTW method and Cox proportional hazards model	16,800 patients, 5,970 (36%) in SR and 10,830 (64%) in ER	AFS	Poor AFS in SR group (HR 1.16, 95% CI: 1.13–1.20) compared with ER.<br><br>No difference in mortality (HR 0.94, 95% CI: 0.89–1.11) between two groups.	Only non-federal California patients included.<br><br>Few variables included in IPTW model - age, sex, race, insurance, diabetes, CAD, and renal failure

© 2022 Majmundar M et al. *JAMA Network Open.*

**Lida et al,[20] 2017**	January 2012 to March 2013	Multicenter, prospective, observational study with propensity-score matching analysis	548 Japanese CLI patients, 197 in SR and 351 in ER initially and 149 in SR and 295 in ER after propensity-score matching	AFS at 3 years	Similar AFS 52% in both groups (P = 0.26)<br><br>Similar 3-year overall survival (57% in SR vs 53% in ER, P = 0.24).<br><br>Similar 3-year limb salvage rate (90% in SR vs 92% in ER, P = 0.82).<br><br>Similar risk of perioperative adverse events (MI 0.7% in SR vs 0.1% in ER, P=0.54; stroke 1.3% in SR vs 1.2% in ER, P=0.93).	Higher percentage (55%) of patients on dialysis<br><br>Novel endovascular devices, including drug-eluting balloons and atherectomy devices were not used
**Kolte et al,[5] 2017**	2013-2014	Retrospective analysis	60 998 patients, 31,685 in ER, 23,172 in SR and 6,141 in the hybrid group.	30-day, all-cause, unplanned readmissions	No significant difference in 30-day readmission rates between SR and ER after adjusting potential confounding variables.	

*AFS, that is, time until major amputation of the index limb and/or death from any cause.
Abbreviations: AFS: amputation-free survival, CAD: coronary artery disease, CI: confidence interval, CLI: critical limb ischemia, CRITISCH: Registry of First-Line Treatments in Patients With Critical Limb Ischemia, ER: endovascular revascularization, HR: hazard ratio, IPTW: inverse probability treatment weighting, MI: myocardial infarction, OSHPD: Office of Statewide Health Planning and Development, SPINACH: Surgical Reconstruction Versus Peripheral Intervention in Patients With Critical Limb Ischemia, SR: surgical revascularization.

© 2022 Majmundar M et al. *JAMA Network Open.*

eFigure. Balance of Covariates Between 2 Revascularization Strategies

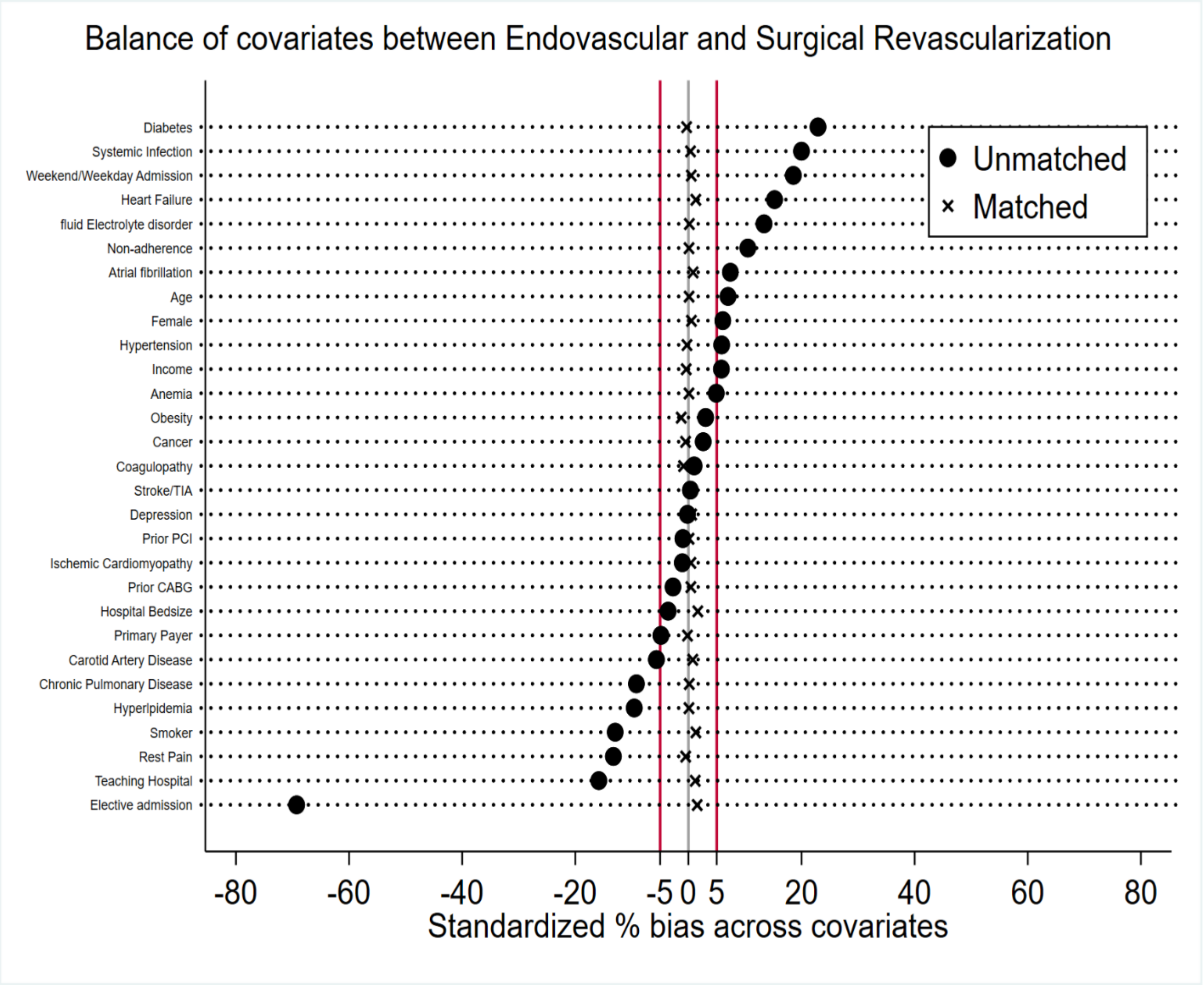

© 2022 Majmundar M et al. *JAMA Network Open.*

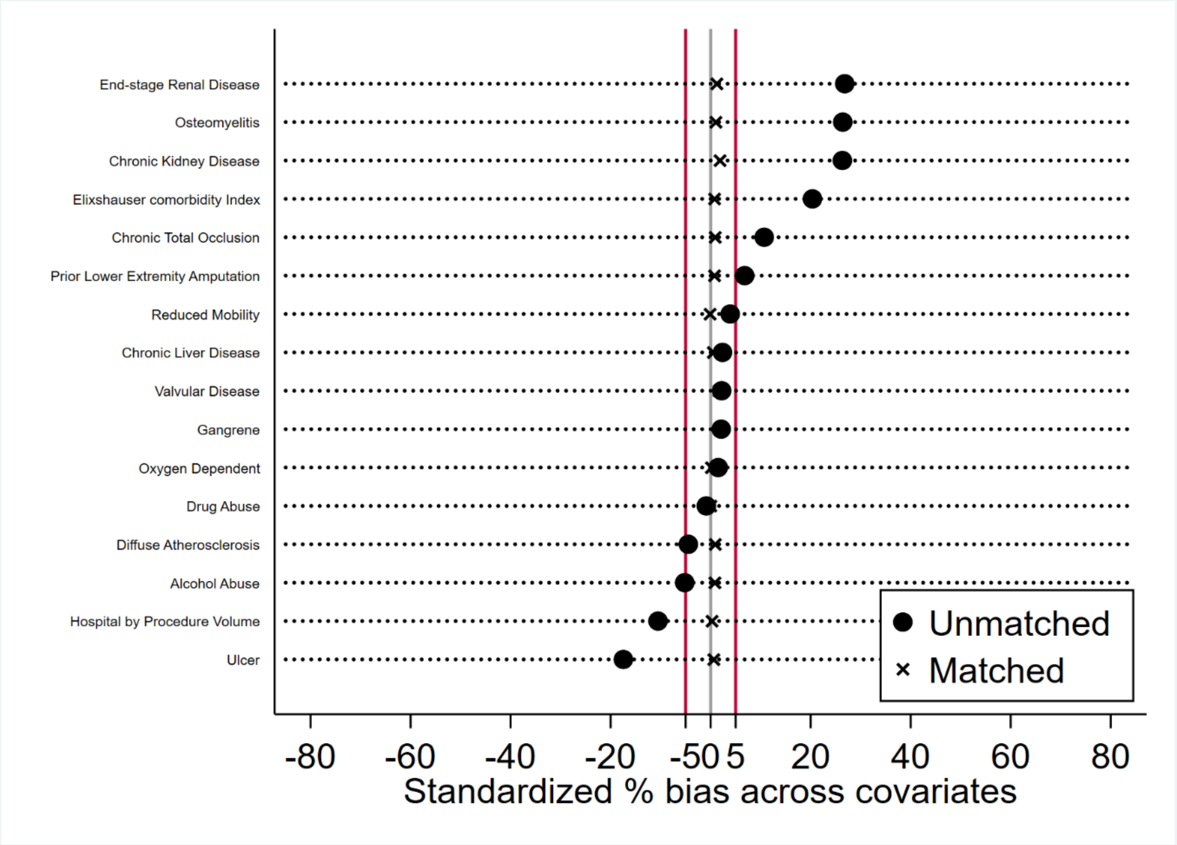

© 2022 Majmundar M et al. *JAMA Network Open.*
